# Single-Molecule Observation of Competitive Protein–Protein Interactions Utilizing a Nanopore

**DOI:** 10.1021/acsnano.4c13072

**Published:** 2024-12-24

**Authors:** Jiaxin Sun, Antun Skanata, Liviu Movileanu

**Affiliations:** † Department of Physics, Syracuse University, 201 Physics Building, Syracuse, New York 13244-1130, United States; ‡ The BioInspired Institute, Syracuse University, Syracuse, New York 13244, United States; § Department of Biomedical and Chemical Engineering, Syracuse University, 329 Link Hall, Syracuse, New York 13244, United States; ∥ Department of Biology, Syracuse University, 114 Life Sciences Complex, Syracuse, New York 13244, United States

**Keywords:** ion channel, protein dynamics, single-molecule electrophysiology, protein engineering, protein hub

## Abstract

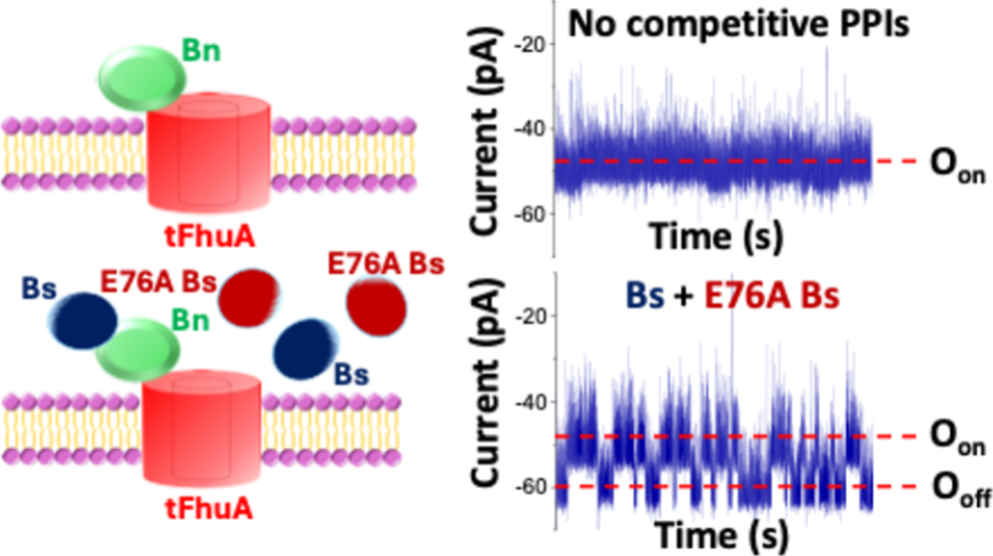

Two or more protein ligands may compete against each other to interact transiently with a protein receptor. While this is a ubiquitous phenomenon in cell signaling, existing technologies cannot identify its kinetic complexity because specific subpopulations of binding events of different ligands are hidden in the averaging process in an ensemble. In addition, the limited time resolution of prevailing methods makes detecting and discriminating binding events among diverse interacting partners challenging. Here, we utilize a genetically encoded nanopore sensor to disentangle competitive protein–protein interactions (PPIs) in a one-on-one and label-free fashion. Our measurements involve binary mixtures of protein ligands of varying binding affinity against the same receptor, which was externally immobilized on the nanopore tip. We use the resistive-pulse technique to monitor the kinetics and dynamics of reversible PPIs without the nanopore confinement, with a high-time bandwidth, and at titratable ligand concentrations. In this way, we systematically evaluate how individual protein ligands take their turn to reside on the receptor’s binding site. Further, our single-molecule determinations of these interactions are quantitatively compared with data generated by a two-ligand, one-receptor queuing model. The outcomes of this work provide a fundamental basis for future developments aimed at a better mechanistic understanding of competitive PPIs. Moreover, they may also form a platform in drug development pipelines targeting high-complexity PPIs mediated by protein hubs.

## 1 Introduction

Protein–protein interactions (PPIs) are the most fundamental and abundant molecular events in cell signaling.^1−3^ Competitive PPIs belong to a subclass of these processes, facilitating the interactions of one protein receptor with multiple protein ligands, one at a time.^4^ While they underlie many biochemical events at the cellular and subcellular levels, the quantitative framework of their kinetics and dynamics has been modestly studied. The discovery and continued explorations of the human proteome have ignited numerous functional studies of competitive PPIs processed by multitasking binding sites^5,6^ of diverse protein hubs. For instance, WD40-repeat protein 5 (WDR5), a nuclear hub involved in regulatory mechanisms of gene expression and cell development, features one binding site that mediates its interactions with dozens of proteins.^7^ Further, c-myelocytomatosis (MYC), an oncoprotein transcription factor and a primary cancer driver, transiently interacts with 336 binding proteins using one of the six evolutionarily conserved MYC homologous boxes.^8−10^ Furthermore, competitive PPIs occur outside the cell. For example, the epidermal growth factor receptor (EGFR) is regulated by its interactions on the extracellular side with several competing growth factor ligand proteins.^11,12^

Many protein ligands of varying affinity and local concentration determine the complex kinetics of cellular and extracellular competitive PPIs. Existing real-time kinetic methods and biochemical assays cannot untangle the complicated distribution of subpopulations of binding events produced by diverse competing protein ligands. The major obstacles to evaluating competitive PPIs include their brief duration and the heterogeneous location of multiple interacting components. Reversible PPIs have been detected using sufficiently large nanopores that facilitated tethered protein receptors on their internal surface.^13,14^ Yet, their interactions with protein ligands may likely be significantly affected by the confinement of the nanopore interior. These technological shortcomings prevent further detailed studies aimed at a better quantitative understanding of competitive PPIs.

**Figure 1:**
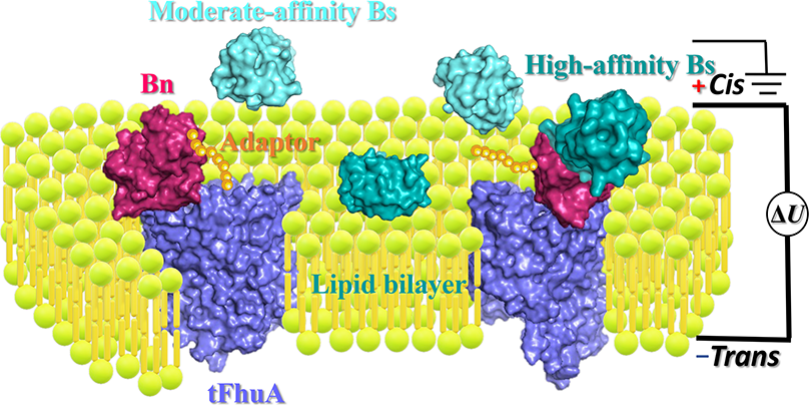
Experimental design for analyzing competitive PPIs using a nanopore. The composition of this single-polypeptide chain protein, Bn–tFhuA, included a folded protein receptor (barnase, Bn; marked in magenta) tethered to the N terminus of a monomeric protein nanopore stem (tFhuA; marked in violet). A dodecapeptide adaptor (marked in dark yellow) was fused at the N terminus of Bn as a reporter of the binding events. Two different protein ligands, a high-affinity ligand (high-affinity Bs (barstar); marked in dark cyan) and a moderate-affinity ligand (moderate-affinity Bs; marked in light cyan), are competitively captured, on a one-on-one basis, at the tip of the nanopore on the *cis* side of a lipid bilayer (in light yellow).

Here, we show that this persistent challenge can be overcome by using a highly specific single-molecule probe approach. We utilize the resistive-pulse technique^15^ and a sensitive nanopore sensor with an external protein receptor. Its binding site is exposed to protein ligands in solution so they can be captured outside the nanopore (Figure 1).

In this study, a transient change in the unitary current readily records each capture and release of the ligand by the tethered receptor. Hence, our nanopore sensor serves for the current readout of the time-resolved protein ligand–protein receptor interactions. Here, the nanopore probe is the transmembrane β barrel^16^ of ferric hydroxamate uptake component A (FhuA)^17,18^ of *Escherichia coli*. This is a 455-residue single-polypeptide chain protein, also called tFhuA.^19^ As a test case, the protein receptor-protein ligand pair is the barnase (Bn)–barstar (Bs) system, respectively.^20^ Bn, a 110-residue RNase,^21^ was fused to the N terminus of tFhuA through a flexible spacer, resulting in a genetically encoded sensor, Bn–tFhuA, for studying the competitive PPIs (Experimental Section). Bs^20^ is the high-affinity 89-residue interacting partner for Bn.^22^ The Bn–Bs complex has been extensively examined as a receptor–ligand model system for electrostatically enhanced PPIs^23^ under numerous experimental conditions and through wide-ranging subsets of mutants.^21,24^ Therefore, the binding interface of each protein has been well characterized.^25^ Further, both proteins are relatively small and highly stable in the aqueous phase.^26^ These features motivated us to postulate that the fusion of Bn to tFhuA does not alter the functional properties of the tethered receptor. In this work, the primary benefit of using the Bn–Bs system is its tractable behavior, showing uniform subpopulations of binding events. This unimodal protein recognition by diverse protein ligands of varying affinity enabled detailed quantitative evaluations of the competitive PPIs.

In this article, we analyze PPIs with only one protein ligand in solution or binary mixtures of protein ligands of varying binding affinity and at titratable concentrations. The resulting change in the unitary current of the binding events is independent of the ligand concentration and its affinity against the tethered receptor. Therefore, this feature makes these current transitions acquired with binary mixtures indistinguishable with respect to a binding event caused by a specific ligand. Here, because we utilize a high-time bandwidth^27^ for the resolvability of competitive PPI events in a one-on-one fashion, our approach permits accurate identifications of their subpopulations generated by individual protein ligands. Specifically, we find that the mean durations of ligand captures are independent of the ligand concentrations but only dependent on the binding affinity against the tethered Bn receptor. This trait was advantageously utilized to assess the contributions of the individual ligands to the receptor occupancy. In addition, this strategy instrumentally helps disentangle the kinetic complexity of the competitive molecular process, even in a simple case when two distinct ligands are present in the solution. For example, we find a nonmonotonic dependence of the receptor occupancy on the competing ligand concentration in binary mixtures with high-affinity and medium-affinity interacting partners. This outcome can be explained using a two-ligand, one-receptor queuing model, in which one ligand requires a waiting time before its turn to reside on the receptor. Such an additional waiting time, which depends on the capture and release durations of the other competing protein ligand of the binary mixture, decreases the receptor occupancy compared to its expected value in the absence of a competitive process.

## 2 Results

### 2.1 Single-Molecule Detection of Transient PPIs Reveals Unimodal Protein Recognition

**Figure 2:**
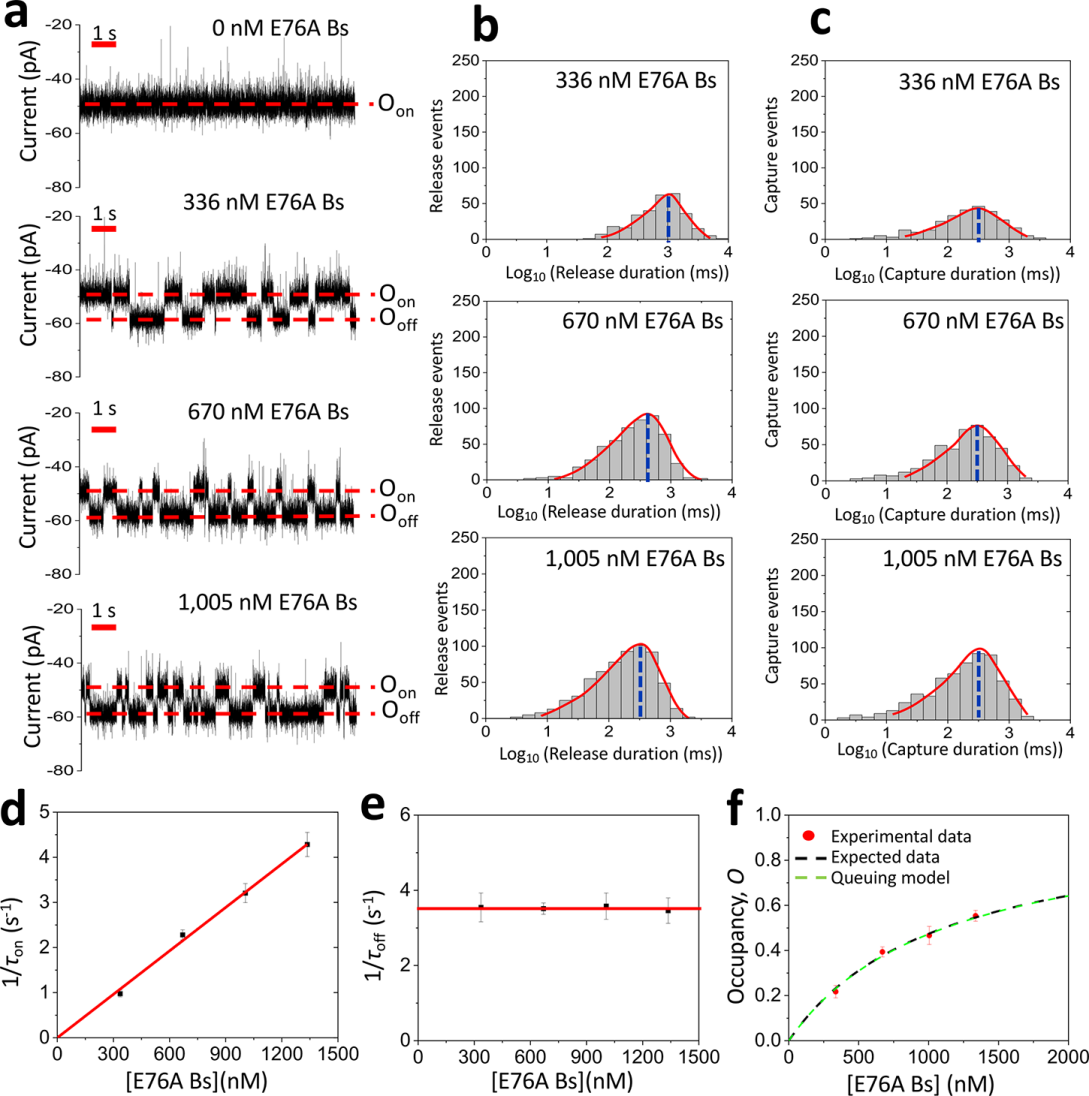
Determination of the Bn–E76A Bs interaction using Bn–tFhuA. (a) Single-channel electrical traces, which were filtered at 1 kHz using a low-pass 8-pole Bessel filter, are provided for 0, 336, 670, and 1005 nM E76A Bs added to the *cis* side of the chamber. The O_on_ and O_off_ levels represent the Bs-released and Bs-captured substates, respectively. The applied transmembrane potential was −40 mV. These traces represent a subset of *n* = 3 distinct functionally reconstituted nanopores. (b) Representative E76A Bs-release duration event histograms at various [E76A Bs] values. The τ_on_ release durations (mean ± s.e.m.) from these histograms were 1013 ± 39 ms (the number of events: *N* = 322), 416 ± 36 ms (*N* = 506), and 309 ± 38 ms (*N* = 657) at 336, 670, and 1005 nM E76A Bs, respectively. (c) Representative E76A Bs-capture duration event histograms at various [E76A Bs] values. The τ_off_ capture durations (mean ± s.e.m.) from these histograms were 313 ± 12 ms (the number of events *N* = 316), 298 ± 17 ms (*N* = 497), and 307 ± 14 ms (*N* = 646) at 336, 670, and 1005 nM E76A Bs, respectively. (d) Diagram illustrating the dependence of 1/τ_on_ on [E76A Bs]. The slope provides a *k*_on_ of (0.32 ± 0.05) × 10^7^ M^–1^ s^–1^. (e) Diagram illustrating the dependence of 1/τ_off_ on [E76A Bs]. The intersection of the horizontal line fit with the vertical axis provides a *k*_off_ of 3.5 ± 0.1 s^–1^. Data points in (d) and (e) represent mean ± s.d. using *n* = 3 independently conducted experiments. (f) The dependence of the receptor occupancy on [E76A Bs]. The expected and experimental occupancies, *O*^Mod^([E76A Bs]) and *O*^Exp^([E76A Bs]), respectively, are determined using eqs 7 and 8) from the Experimental Section.

First, we examined the signature of Bn–tFhuA without and with individual protein ligands. Then, complex single-channel electrical signatures were recorded in binary mixtures of competing protein ligands of varying binding affinity and at titratable concentrations. Bn–tFhuA exhibited a quiet electrical signature with an average conductance of 1.22 ± 0.04 nS (*n* = 5) in 300 mM KCl, 10 mM Tris-HCl, and pH 8.0 at an applied transmembrane potential of −40 mV (Figure 2a), demonstrating that the tFhuA nanopore tolerates large polypeptide extensions on its N terminus without deterioration in its pore-forming properties.^19^ A dense cluster of ion-pair contacts mediates the high-affinity Bs–Bn interaction.^28,29^ Selective substitutions of charged residues among key ion pairs of the Bs–Bn binding interface may significantly alter the affinity.^24,30^ Therefore, this feature can produce Bs protein ligands with varying binding characteristics.

Here, we used three protein ligands: a high-affinity Bs, a medium-affinity E76A Bs, and a weak-affinity D39A Bs. Next, we show an example of the single-molecule data with the medium-affinity ligand. E76A Bs added to the *cis* side produced reversible current transitions, whose amplitude was independent of E76A Bs concentration, [E76A Bs] (Figures 1, 2a; Supporting Information Figures S1–S3, and S4ab, and Table S1). The O_on_ and O_off_ current levels correspond to the ligand-released and ligand-captured substates, respectively. The average conductance of the ligand-captured substate was 1.50 ± 0.05 nS (*n* = 5). The slight increase of ∼0.28 nS upon ligand binding is intriguing. Notably, the conductance value corresponding to the ligand-captured substate is closely like that measured with tFhuA (∼1.52 nS),^31^ which does not contain the tethered Bn receptor. Because the ligand-released substate corresponded to a declined conductance level, we interpret that Bn, along with the N-terminal peptide adaptor (Figure 1, Experimental Section), likely adopts an orientation that partly blocks the ionic flow near the nanopore opening. Upon ligand binding, this specific orientation is significantly altered, leading to a full recovery of the ionic flow. This interpretation is also supported by a noisier, reduced-current O_on_ substate than a quieter increased-current O_off_ substate (Figure 2a).

We utilized the maximum likelihood method^32^ and logarithm likelihood ratio (LLR)^33−35^ tests to compare different fitting models for event duration histograms. This way, the best models of the probability distribution functions (PDFs) were determined. At a confidence level *C* = 0.95, a single-exponential fit was the best model for the protein ligand-released and protein ligand-captured mean durations, the τ_on_ and τ_off_ time constants, respectively. Fits to a two-exponential model were not statistically superior, as judged by the LLR value.

In a semilogarithmic representation, the event histograms of the E76A Bs-released durations (Figure 2b; Supporting Information Figure S4c) and E76A Bs-captured durations (Figure 2c; Supporting Information Figure S4d) revealed single-exponential distribution of time constants. It should be noted that the logarithm of the time constant is the center location of the histogram peak. The main advantage of such representations is the improved fit quality for two or more widely separated subpopulations in terms of the time constant (see below).^36^ This benefit is helpful when comparing the dynamic change of distinct distributions of binding durations produced by competing protein ligands. Therefore, they are a practical alternative to nonlogarithmic representations of dwell time histograms (Supporting Information Figure S5) or scatter plots of ligand-released and ligand-captured durations.

At increased [E76A Bs] values, τ_on_ declined, meaning an increase in the frequency of binding events (Figure 2a,b; Supporting Information Table S2). Moreover, the frequency of E76A Bs-captured events, in the form of the reciprocal of the protein ligand-released duration (1/τ_on_), increased linearly and in a ratio ∼1:1 with [E76A Bs] (Figure 2d), indicating a bimolecular association process between the tethered Bn receptor and the E76A Bs protein ligand. The slope of the linear fit of the event frequency was the association rate constant (*k*_on_). Yet, τ_off_ was independent of the [E76A Bs] value, confirming the unimolecular dissociation process. The reciprocal of τ_off_ is the dissociation rate constant (*k*_off_; Figure 2e). We obtained *k*_on_ = (0.32 ± 0.05) × 10^7^ M^–1^ s^–1^ and *k*_off_ = 3.5 ± 0.1 s^–1^, yielding an equilibrium dissociation constant *K*_D_ of 1.1 ± 0.1 μM (Supporting Information Table S3). Finally, these kinetic constants enabled the evaluation of the [E76A Bs]-dependent receptor occupancy, *O* (Figure 2f, the Experimental Section, eqs 7 and 8), given by the ratio of the total ligand-bound duration to the total recording time. These experiments demonstrate single-exponential distributions of binding durations, resulting in unimodal protein recognition. This finding contrasts with transient PPIs that obey a multimodal protein recognition given by distinct subpopulations of binding events.^31,37^

### 2.2 Competitive Reversible PPIs of High- and Moderate-Affinity Protein Ligands

**Figure 3:**
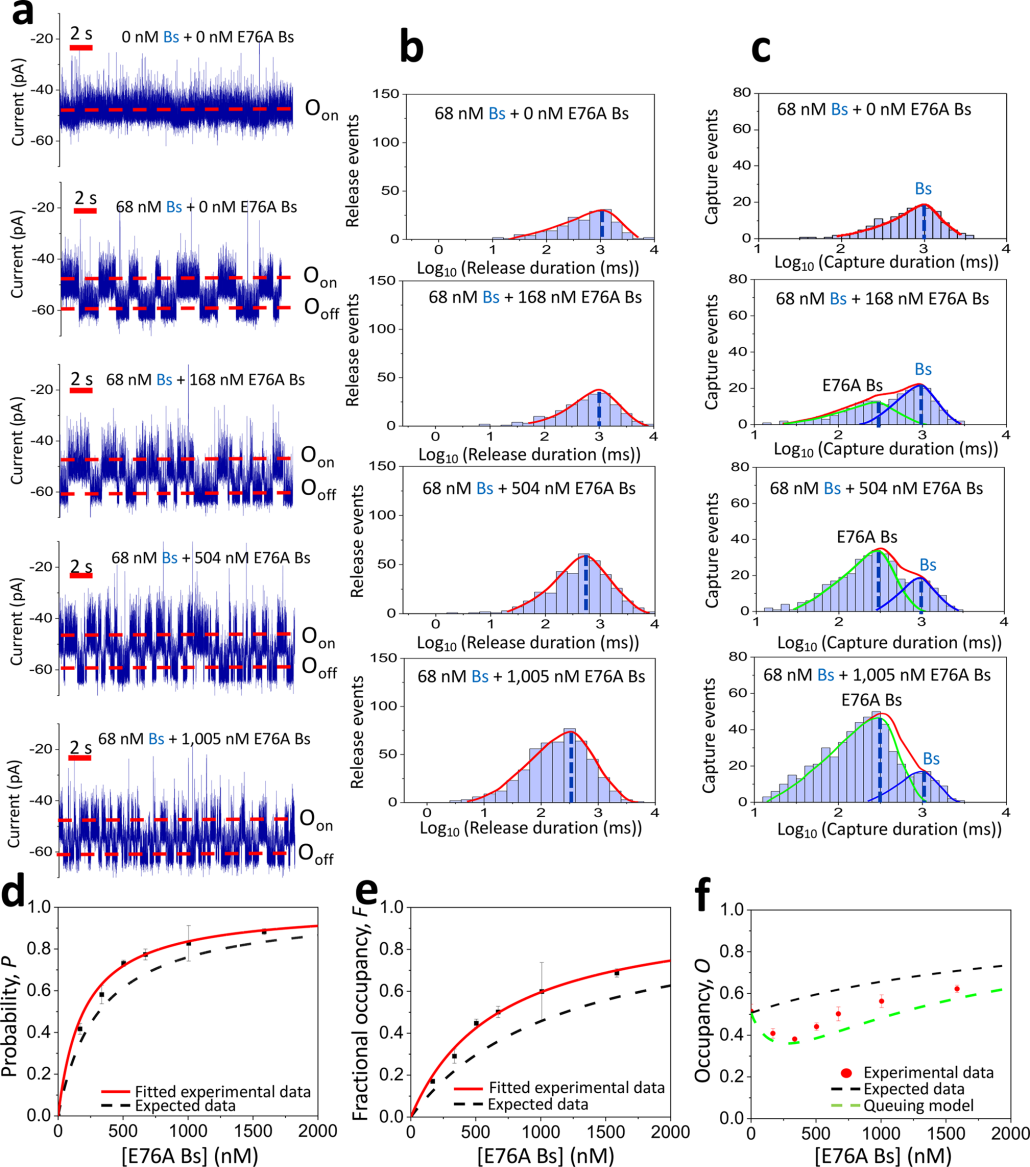
Competitive PPIs of strongly and moderately binding interactions. (a) Representative single-channel electrical traces were filtered at 1 kHz using a low-pass 8-pole Bessel filter for binary mixtures of strongly and moderately binding protein ligands. The protein binary mixture included 68 nM Bs and various [E76A Bs] values added to the *cis* side of the chamber. The O_on_ and O_off_ levels represent the ligand-released and ligand-captured substates, respectively. The applied transmembrane potential was −40 mV. These single-channel electrical traces are representative of a subset of *n* = 3 distinct nanopores. (b) Representative semilogarithmic duration histograms of ligand-released events at various [E76A Bs] values. The red curves represent the cumulative fits for the ligand-released durations. The τ_on_ release durations (mean ± s.e.m.) from these histograms were 977 ± 44 ms (the number of events: *N* = 164), 879 ± 57 ms (*N* = 203), 556 ± 41 ms (*N* = 357), and 315 ± 37 ms (*N* = 519) at [E76A Bs] values of 0, 168, 504, and 1005 nM, respectively. (c) Representative semilogarithmic duration histograms of ligand-captured events at various [E76A Bs] values. The red curves represent the cumulative fits for the ligand-released durations. The green and blue curves represent the composite peak fits for the E76A Bs- and Bs-captured event durations, respectively. The τ_off_ capture durations (mean ± s.e.m.) from these histograms were 1031 ± 56 ms (the number of events: *N* = 154), 282 ± 23 and 912 ± 47 ms (*N* = 203), 276 ± 28 and 933 ± 39 ms (*N* = 350), and 291 ± 42 and 956 ± 62 ms (*N* = 519) at [E76A Bs] values of 0, 168, 504, and 1005 nM, respectively. (d) The dependence of the probability, *P*, of E76A Bs-captured binding events on the [E76A Bs] value. The black dashed line represents the expected data based on the *k*_on_ generated from the individual Bn–Bs and Bn–E76A Bs binding assays (*k*_on-E76A Bs_ = 0.32 × 10^7^ M^–1^ s^–1^, *k*_on-Bs_ = 1.48 × 10^7^ M^–1^ s^–1^). The red continuous line represents the fitted experimental data. Using the fit of experimental data (eq 3), the *k*_on_ for E76A Bs and Bs (mean ± s.e.m.) were (0.49 ± 0.04) × 10^7^ M^–1^ s^–1^ and (1.32 ± 0.08) × 10^7^ M^–1^ s^–1^, respectively. (e) Diagram illustrating the dependence of the fractional occupancy, *F*, of E76A Bs-captured binding events on the [E76A Bs] value. The dashed line represents the model data based on the *K*_D_ generated from the individual Bn–Bs and Bn–E76A Bs binding assays (*K*_D-Bs_ = 64 nM and *K*_D-E76A Bs_ = 1.1 μM). The red continuous line represents the fit of experimental data. (f) Diagram illustrating the dependence of the experimental and expected receptor occupancies, *O*, at various [E76A Bs] values. These values are determined using eqs 7 and 8) (Experimental Section).

Next, we examined binary mixtures of protein ligands of varying binding affinity at titratable concentrations. In the first subset of experiments, these binary mixtures included high- and moderate-affinity ligands, namely Bs and E76A Bs, respectively. Long-lived current transitions were noted when 68 nM Bs, a high-affinity protein ligand, was added to the *cis* side (Figure 3a, the top two traces). The Bs-released and Bs-captured durations were 995 ± 59 and 1057 ± 85 ms, respectively (*n* = 3; Supporting Information Table S4), yielding an equilibrium dissociation constant *K*_D_ of ∼64 nM. Next, [E76A Bs] was increased at various levels while [Bs] was kept at 68 nM. The current amplitudes of protein ligand-captured events were uniformly distributed over a Gaussian peak regardless of [E76A Bs]. This finding suggests the indistinguishability of Bs- and E76A Bs-captured current substates (Figure 3a; Supporting Information Figures S6, S7 and Table S5).

However, a time-based data analysis utilizing LLR tests^33−35^ revealed single- and two-exponential distributions of the ligand-released and ligand-captured durations, respectively (Figure 3b,c; Supporting Information Figure S8). Interestingly, the two peaks of ligand-captured events featured maxima, whose center locations corresponded to mean durations closely like binding times of [Bs] and [E76A Bs] (Supporting Information Table S4). Therefore, the mean durations of these subpopulations of binding events indicate that they correspond to Bs-captured (τ_off-Bs_) and E76A Bs-captured (τ_off-E76A Bs_) events. Using the event frequency of Bs-captured (*f*_Bs_) and E76A Bs-captured (*f*_E76A Bs_) binding events as well as the ligand-released duration (τ_on_), we determined the individual Bs-released and E76A Bs-released durations, τ_on-Bs_ and τ_on-E76A Bs_, respectively (Supporting Information Table S6).*f*_Bs_ and *f*_E76A Bs_ were used further to determine the experimental probability, *P*_E76A Bs_^Exp^, of the varying protein ligand concentration in solution (Experimental Section, eq 3; Figure 3d; Supporting Information Tables S7 and S8). Assuming that the mean durations of ligand-captured events are independent of [E76 A Bs] (Supporting Information Table S4) and utilizing the *k*_on_ values derived from the individual, noncompetitive Bn–Bs and Bn–E76A Bs binding assays (Experimental Section, eq 4), we inferred the expected probability of E76A Bs events, *P*_E76A Bs_^Mod^, which was slightly lower than *P*_E76A Bs_^Exp^ (Figure 3d).

Using the time constants of individual capture and release durations enabled the determination of rate constants corresponding to each protein ligand. The association rate constants of Bs and E76A Bs, *k*_on-Bs_ and *k*_on-E76A Bs_ (mean ± s.e.m.), were (0.79 ± 0.09) × 10^7^ M^–1^ s^–1^ and (0.26 ± 0.03) × 10^7^ M^–1^ s^–1^, respectively (Supporting Information Table S6). The corresponding dissociation rate constants of the same interactions, *k*_off-Bs_ and *k*_off-E76A Bs_ (mean ± s.e.m.), were 1.1 ± 0.1 and 3.7 ± 0.1 s^–1^, respectively (Supporting Information Table S4). These values yielded the corresponding *K*_D_ values for Bs and E76A Bs of 141 ± 14 nM and 1.5 ± 0.2 μM, respectively. We defined the fractional occupancies, *F*, of the tethered Bn receptor by the total time of a specific ligand-occupied receptor normalized to the total occupied time of the receptor. As expected, the experimental fractional occupancy made by E76A Bs, *F*_E76A Bs_^Exp^, increased by enhancing [E76A Bs] in solution (Figure 3e). Using the fit of *F*_E76A Bs_^Exp^, we found that the *K*_D_ values for Bs and E76A Bs were 101 ± 9 nM and 0.97 ± 0.02 μM, respectively. This outcome is in accordance with the corresponding *K*_D_ values determined from noncompetitive assays of Bn–Bs and Bn–E76A Bs interactions, respectively (Supporting Information Table S3 and S6). Utilizing the same assumption as above and the *K*_D_ values generated from the individual Bn–Bs and Bn–E76A Bs binding assays (Experimental Section, eq 6), the expected fractional occupancy, *F*_E76A Bs_^Mod^, was determined and followed the same pattern with the experimental value (Supporting Information Figure S9).

### 2.3 Biphasic Dependence of the Experimental Receptor Occupancy on the Competing Protein Ligand

Next, we calculated the experimental occupancy of the receptor, *O*^Exp^, as the total ligand-occupied time normalized to the total recording time (Figure 3f). Surprisingly, a biphasic pattern was noted by elevating [E76A Bs] (Supporting Information Tables S7 and S8). At low [E76A Bs] values, *O*^Exp^ was lower with respect to the baseline value of ∼0.5, which was expectedly acquired at a Bs concentration, [Bs], near its *K*_D_. Then, the occupancy increased monotonously at greater [E76A Bs] values. This interesting concentration dependence of *O*^Exp^ at low [E76A Bs] was likely determined by the balance of a relative reduction in the Bs-captured events with a relatively longer mean duration of 930 ± 10 ms and an increase in the E76A Bs-captured events with a relatively shorter mean duration of 272 ± 6 ms (*n* = 3 independently reconstituted nanopores; Supporting Information Table S4). Beyond 336 nM E76A Bs, the varying ligand dominated the receptor occupancy. It should be noted that the expected occupancy, *O*^Mod^, given kinetic parameters acquired from noncompetitive binding assays (Experimental Section, eq 7), exhibits a monotonic dependence on [E76A Bs]. This discrepancy between the experimental and expected values suggests a subtle mechanism of the competitive PPIs that is detectable in our single-molecule determinations.

### 2.4 Competitive PPIs of High- and Weak-Affinity Protein Ligands

**Figure 4:**
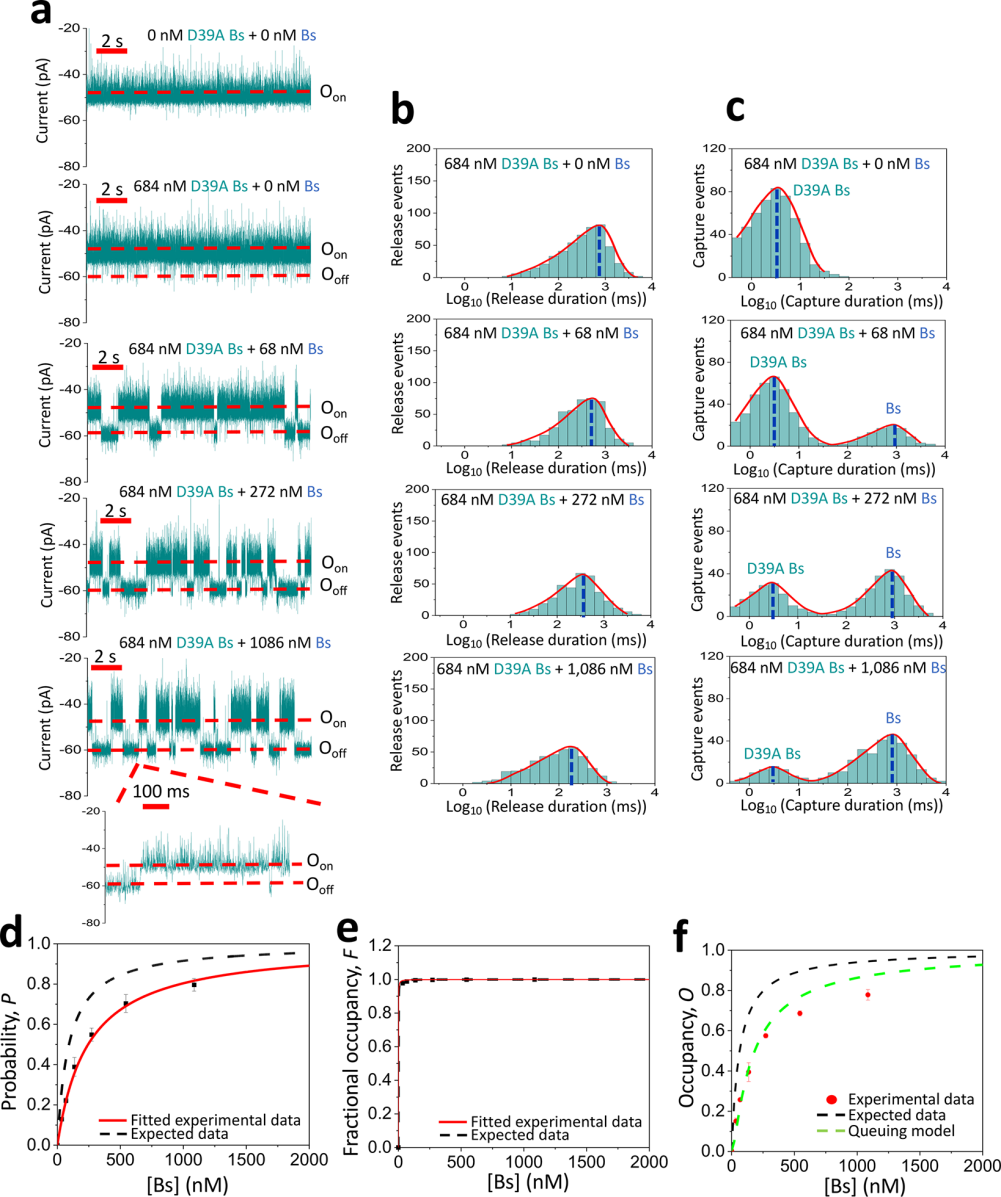
Competitive PPIs with weakly and strongly binding interactions. (a) Single-channel electrical traces are provided for binary mixtures of weakly and strongly binding protein ligands. These binary mixtures contained 684 nM D39A Bs and a varying concentration of Bs added to the *cis* side of the chamber. The O_on_ and O_off_ levels correspond to the ligand-released and ligand-captured substates, respectively. The applied transmembrane potential was −40 mV. These single-channel electrical traces are representative over a subset of *n* = 3 distinct nanopores. (b) Representative semilogarithmic duration histograms of ligand-released events at various [Bs] values. The τ_on_ release durations (mean ± s.e.m.) from these histograms were 708 ± 37 ms (the number of events: *N* = 486), 550 ± 29 ms (*N* = 442), 342 ± 21 ms (*N* = 367) and 162 ± 13 ms (*N* = 370), at 0, 68, 272, and 1086 nM Bs, respectively. (c) Representative semilogarithmic duration histograms of ligand-captured events at various [Bs] values. The τ_off_ capture durations (mean ± s.e.m.) from these histograms were 3.3 ± 0.1 ms (the number of events: *N* = 486), 2.9 ± 0.1 and 871 ± 56 ms (*N* = 442), 2.8 ± 0.1 and 851 ± 29 ms (*N* = 367), and 2.8 ± 0.1 and 837 ± 41 ms (*N* = 370) at 0, 68, 272, and 1086 nM Bs, respectively. In panels (c) and (d), the red curves represent the cumulative fits for the ligand-released and ligand-captured durations, respectively. (d) Diagram illustrating the dependence of the probability, *P*, of Bs-captured binding events on the [Bs] value. The black dashed line represents the expected data based on the *k*_on_ generated from the individual Bn–Bs and Bn–D39A Bs binding assays (*k*_on-Bs_ = 1.48 × 10^7^ M^–1^ s^–1^ and *k*_on-D39A Bs_ = 0.20 × 10^7^ M^–1^ s^–1^). The red, thick, and continuous line represents the fitted experimental data. Using the fit of experimental data (eq 4), the *k*_on_ for Bs and D39A Bs were (1.05 ± 0.02) × 10^7^ M^–1^ s^–1^ and (0.37 ± 0.01) × 10^7^ M^–1^ s^–1^, respectively. (e) Diagram showing the dependence of the fractional occupancy, *F*, of Bs-captured binding events on the [Bs] value. The black dashed line represents the expected data based on the *K*_D_ generated from the individual Bn–Bs and Bn–D39A Bs binding assays (*K*_D-Bs_ = 64 nM and *K*_D-D39A Bs_ = 168 μM). The red, thin, and continuous line represents the fitted experimental data. Using the fit of experimental data (eq 6), the *K*_D_ for D39A Bs and Bs were 187 ± 14 μM and 63 ± 2 nM, respectively. (f) Diagram illustrating the dependence of the occupancy, *O*, of the Bn binding site at various [Bs] values. These values are determined using eqs 7 and 8 from the Experimental Section.

The kinetics of competitive PPI radically change when the binary mixture includes weakly and strongly binding protein ligands. Let us consider that the weakly binding D39A Bs protein will be kept at a constant concentration of 684 nM. Then, the strongly binding Bs concentration, [Bs], will be titrated on the *cis* side. The D39A Bs and Bs produced very brief and long-lived current transitions (Figure 4a; Supporting Information Figure S10). Again, these transitions featured a current amplitude scattering within the same Gaussian peak (Supporting Information Table S9 and Figures S10, S11), suggesting similar binding mechanisms of the two protein ligands with the tethered Bn receptor. In addition, this result rules out the possibility of nonspecific binding of D39A Bs to the receptor, which otherwise would generate a different current transition signature. As in the previous case, the LLR tests analysis revealed single- and two-peak distributions of the ligand-released and ligand-captured durations, respectively (Figure 4b,c; Supporting Information Figure S12). The mean durations of individual Bs- and D39A Bs-captured events, τ_off-Bs_ and τ_off-D39A Bs_, respectively, were independent of [Bs]. In contrast, the interevent duration, τ_on_, gradually decreased at elevated [Bs] levels (Supporting Information Table S10).

The association rate constants of the two probed competitive interactions, *k*_on-Bs_ and *k*_on-D39A Bs_ (mean ± s.e.m.) were (0.53 ± 0.08) × 10^7^ M^–1^ s^–1^ and (0.18 ± 0.02) × 10^7^ M^–1^ s^–1^, respectively (Supporting Information Table S11). The same competitive PPIs for the Bs–Bn and D39A Bs–Bn interactions exhibited the dissociation rate constants (mean ± s.e.m.) of 1.2 ± 0.1 and 351 ± 9 s^–1^, respectively (Supporting Information Table S10). The corresponding *K*_D_ values were 229 ± 45 nM and 200 ± 32 μM, respectively. Therefore, our approach can be utilized to concurrently discriminate competitive PPIs that differ by 3 orders of magnitude from each other. The probability, *P*_Bs_([*Bs*]), and fractional occupancy, *F*_Bs_([*Bs*]), were anticipatedly amplified at elevated [Bs] (Figure 4d,e; Supporting Information Tables S12 and S13). Because of a relatively long Bs-captured duration, τ_Bs_, *F*_Bs_([*Bs*]) almost immediately rose to the maximum value. In contrast to the above-mentioned case, the experimental occupancy, *O*^Exp^, showed an increasing value throughout the [Bs] range examined in this study (Figure 4f).

### 2.5 Quantitative Analysis of Competitive PPIs and Queuing Theory

In both examples discussed above, we experimentally and analytically showed that the event probability and fractional receptor occupancy depend on several parameters, such as the kinetic and affinity parameters, as well as the ligand concentrations. Further, we evaluated the expected and experimentally acquired values of the receptor occupancies under different experimental conditions. The biphasic pattern of *O*^Exp^ for the Bs-E76A Bs binary mixture was unexpected at lower concentrations of the moderate-affinity protein ligand. In addition, *O*^Exp^ was always lower than the expected occupancy, *O*^Mod^, of the same binary mixture and under similar experimental conditions (Figure 3f). The latter finding was also replicated with the Bs-D39A Bs binary mixture (Figure 4f), suggesting a closely related mechanism involved in the competitive PPIs that makes our predicted values of the receptor occupancy overestimated. Hence, we hypothesize that a queuing process occurs when protein ligands compete to bind against the tethered Bn receptor. This would decline the experimental occupancy with respect to the expected values without a competing PPI process (Supporting Information Tables S8 and S13).

To test this hypothesis, we formulated a simple queuing model with a receptor that shares the same binding site with two competing ligands (Experimental Section, eqs 9–20). This approach involved a probabilistic analysis of the events resulting from waiting lines. For the Bs-E76A Bs binary mixture, the resulting data of the queuing model-based occupancy is plotted in Figure 3f. Remarkably, our analytic result of the two-ligand, one-receptor queuing model reproduces the experimental pattern determined from the single-molecule detection of competitive PPIs. The experimental value of the receptor occupancy reached a minimum (mean ± s.d.) of 0.382 ± 0.004 at a 336 nM E76A Bs (Supporting Information Table S8). Considering the kinetic rate constants of individual PPIs and the unmodified protein ligand concentration (*k*_on-Bs_ = 1.48 × 10^7^ M^–1^ s^–1^, *k*_on-E76A Bs_ = 0.32 × 10^7^ M^–1^ s^–1^, *k*_off-Bs_ = 0.95 s^–1^, *k*_off-E76A Bs_ = 3.6 s^–1^, [Bs] = 68 nM), the two-ligand, one-receptor queuing model would estimate a receptor occupancy of 0.366 at 288 nM E76A Bs. Therefore, the anticipated value of the receptor occupancy independently determined from the queuing model is in accord with the experimental value. For the Bs-D39A Bs binary mixture, the queuing model-based occupancy data is illustrated in Figure 4f. The queuing-model occupancy data with no free fit parameters aligned well with the experimental values, especially in the 0–272 nM Bs concentration range. For this binary mixture, no minimum in the receptor occupancy was experimentally detected.

One immediate question is whether specific experimental conditions can be found for the monotonic and biphasic dependences of the receptor occupancy on the competing ligand concentrations. Using a two-ligand, one-receptor queuing model, we find that the occupancy always shows a biphasic pattern with a minimum located at a critical [L_2_]* concentration of the ligand (Experimental Section, eq 19). The minimum occupancy value, *O*_min_, is given by eq 20 (Experimental Section). For the parameters of the Bs-D39A Bs binary mixture, *k*_on-Bs_ = 1.48 × 10^7^ M^–1^ s^–1^, *k*_on-D39A Bs_ = 0.2 × 10^7^ M^–1^ s^–1^, *k*_off-Bs_ = 0.95 s^–1^, *k*_off-D39A Bs_ = 327 s^–1^, [D39A Bs] = 684 nM, we obtained an *O*_min_ value of 0.004 at a critical [Bs]* value of 0.13 nM. This minimum occupancy was not readily detectable in our Bs titratable range because the biphasic pattern occurs at a subnanomolar concentration of the competing protein ligand. Finally, we explored the Bs-E76A Bs binary mixture with [Bs] maintained at 17 nM. A reduced [Bs] value significantly increased probabilities and fractional occupancies, yet this inhibits the receptor occupancies (Supporting Information Figures S13–S17 and Tables S14–S17). For this experimental condition, *k*_on-Bs_ = 1.48 × 10^7^ M^–1^ s^–1^, *k*_on-E76A Bs_ = 0.32 × 10^7^ M^–1^ s^–1^, *k*_off-Bs_ = 0.95 s^–1^, *k*_off-E76A Bs_ = 3.6 s^–1^, [Bs] = 17 nM. The two-ligand, one-receptor queuing model predicts an *O*_min_ value of 0.137 at 86 nM E76A Bs, which is in good accordance with the experimental *O*_min_ value of 0.165 at 28 nM E76A Bs (Supporting Information Table S17).

The kinetic rate constants of association and dissociation and binding affinities of the ligand–receptor complexes determined in this single-molecule study align with previously measured values of the same parameters.^24^ For example, the *K*_D_ values of Bn interacting with Bs, E76A Bs, and D39A Bs measured in this work using 300 mM KCl were 64 nM, 1.1 μM, and 168 μM, respectively (Supporting Information Tables S3, S6, and S11). Earlier kinetic measurements of the same complexes measured in 50 mM Tris-HCl identified a *K*_D_ of 0.32, 3.5, and 39 nM, respectively.^24^ It should be noted that the latter salt condition belongs to the electrostatic energy–driven interaction regime, whereas our salt concentration corresponds to the thermally driven interaction regime.^23,38^ The substantial decline in the binding affinity at an increased KCl is more than likely due to the extensive screening of the electrostatic interactions at the ligand–receptor interface. Under this condition, the Debye screening length, λ_D_, is shorter than the Bjerrum length, *l*_B_. Here, λ_D_, the range of the electrostatic energy between K^+^ and Cl^–^ at an electrolyte concentration, *I*, and at 25 °C, is^38^
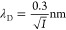
1*l*_B_ (∼0.71 nm), the distance between K^+^ and Cl^–^ at which the electrostatic interaction energy and the thermal energy balance each other, is
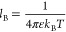
2ϵ, *k*_B_, and *T* are the electric permittivity, Boltzmann’s constant, and absolute temperature, respectively. The electrostatic energy is dominant at *l*_B_ < λ_D_. Using eqs 1 and 2, the boundary between the two regimes occurs at 178.5 mM KCl.

## 3 Discussion

In a recently reported work,^10^ we used a nanopore sensor with an external peptide ligand of MYC against WDR5 via the WDR5 binding motif site (MYC_WBM_).^39^ Single-channel electrical recordings were employed at an amplified single-to-noise ratio to demonstrate short-lived and unimodal captures of WDR5, confirming earlier evidence for this clinically significant weak-affinity interaction.^9,40,41^ Uniquely, that study has provided a new approach to quantitatively probe interactions mediated by different binding sites of the same protein hub using a similar nanopore architecture. For example, the same method can be utilized to identify multimodal protein recognition of WDR5 by a mixed lineage leukemia (MLL) peptide ligand through the WDR5 interaction (Win) site,^42^ which was observed via distinct subpopulations of binding events.

In this study, we use a genetically encoded sensor as a single-polypeptide chain nanopore with a tethered small protein. We examined how the tethered receptor specifically and selectively interacts with competing ligands from binary mixtures at titratable concentrations. Here, the modulation in the ligand affinity was achieved via key mutations in its binding site, producing substantial differences in the capture durations among the competing ligands. Our method is fully quantitative, so these competitive PPIs are evaluated in terms of the event probability and fractional occupancy of a given ligand and the overall receptor occupancy. The test case for these interactions is advantageous because it exhibits uniform and time-resolved subpopulations of binding events attributed to specific ligands. In addition, single-molecule electrical recordings of these binding events are probed using simple data analysis algorithms. The amplitudes of the ligand-captured transitions made by different protein ligands were closely similar, suggesting that distinguishing different ligands using single-channel currents is challenging. In contrast, each protein ligand produced a specific subpopulation of binding event durations, indicating a monomodal protein recognition for each ligand. This was also facilitated by semilogarithmic representations of ligand-captured durations, resulting in accurate evaluations of the number of binding events made by each ligand. Hence, our measurements revealed the kinetic complexity of competitive PPIs that existing technologies cannot identify in the bulk phase due to the averaging process of determinations in an ensemble.^43^

Using queuing theory, we formulate a two-ligand, one-receptor model, accounting for and reproducing the receptor occupancy. Further, this model can be used to predict the existence of the nonmonotonic dependence of the occupancy on the available concentration of the competing protein ligand. Yet, previously formulated analytical models of competitive PPIs either predicted^44^ or illustrated^44−46^ a monotonic dependence of the receptor occupancy on competing ligand concentrations. It should be noted that our model does not include additional physical or structural constraints of the ligand–receptor interactions.^47^ For example, the receptor was tethered to the nanopore via a flexible peptide spacer.

Given the complexity of the networks of PPIs mediated by protein hubs, predicting the experimental occupancy of competitive PPIs by interactions with numerous protein ligands is challenging without an analytical formulation. Our two-ligand, one-receptor queuing model is generalizable to one receptor and multiple ligands to evaluate nontrivial aspects and behaviors of competitive PPIs with numerous ligands. It can also be extended to a receptor with multiple binding sites with or without allosteric regulation. These analytical and computational developments are ongoing in this research group. They may also help form a framework to assess the effects of targeted PPI inhibitors subjected to a protein hub and complex distributions of interacting protein ligands of varying affinity. Tansey and co-workers (2021) have employed quantitative proteomics to demonstrate that an inhibitor of the multitasking Win site of WDR5 drastically alters its interaction with dozens of proteins, some of which are key players in signaling.^7^ These changes in competitive native PPIs are expected to modify a subset of the WDR5 functions,^48^ highlighting the significance of a better understanding of the complex implications of using competitive PPIs’ inhibitors. Therefore, more high-throughput technologies for the comprehensive analysis of proteomic profiling of specific interactomes are in pressing demand.

Our approach contrasts with most nanopore studies that sense molecules by their entry into the pore interior, as extensively reviewed earlier.^49−52^ In the vast majority of these studies, the partitioning of the molecules into the nanopores is a direct way to produce the modulation in the unitary current upon binding to a specific recognition element, interacting with or translocating through the nanopore. This strategy has been proven productive in numerous examples using small organic and inorganic molecules and various biopolymers, such as peptides, nucleic acids, and polysaccharides. Because folded proteins are typically larger than the cross-sectional diameter of nanopores, this approach is less practical for probing reversible PPIs. Moreover, the confinement of the nanopore interior would induce further physical restraints on these interactions. However, larger diameter nanopores have been recently utilized to assess PPIs within their interior.^53^ In this study, we used a redesigned nanopore with a small protein receptor on its external side. This way, competitive PPIs can be monitored externally using the current modulation resulting from ionic flow alterations near the pore entrance. There is no fundamental difficulty in substituting the tethered Bn protein with another receptor and no technical challenge in replacing cognate ligands with other interacting partners. For example, we recently showed that such a redesign can be generalizable to other small proteins, including antibody-mimetic scaffolds for protein biomarker detection.^31^ While our strategy can be employed in other receptor–ligand pairs, potential challenges may arise. For example, larger folded proteins may likely create further steric constraints, precluding the clearance of the region around the pore entrance. In addition, the binding surface of the receptor must be accessible to ligands navigating around the nanopore. Although both methods rely on modulating the unitary current, the former is more sensitive to the voltage drop across the nanopore’s central axis.

tFhuA tolerates large polypeptide extensions on its N-terminus without impairing pore-forming properties. This feature highlights the superior properties of tFhuA, including its robustly folded structure and ease of site-specific protein design without challenges associated with the multimeric nature of pore-forming toxin-based sensors. Yet, one limitation of this nanopore stem is its high hydrophobicity, requiring its renaturing in urea and slow dialysis-based refolding in detergents. Sensing proteins outside nanopores without unfolding them has several advantages, such as detecting different binding sites in their natively folded forms and post-translation modifications (PTMs).^54^ This strategy may ignite further opportunities for monitoring multimodal protein recognition of hubs by specific peptide ligands. Our method may be developed further to probe selectively protein ligands with varying PTMs of their interaction sites, as revealed by distinctive kinetics.^55,56^ From a practical point of view, this method of evaluating competitive PPIs with numerous coexistent ligands may impact the drug discovery pipelines by creating a platform and tools to assess the inhibitory effects of small-molecule compounds in a more realistic, complex, and quantitative formulation.

## 4 Experimental Section

### 4.1 Cloning and Mutagenesis of the Nanopore Sensor and Protein Ligands

Standard and assembly PCR protocols were utilized to develop all genes employed in this study, which were cloned into the pPR-IBA1 expression vector.^17^ An H102A mutant of barnase (Bn) was used because this variant suppressed RNase activity.^21,24^ For the sake of simplicity, we use the Bn nomenclature for this barnase mutant throughout the article. Bn–tFhuA did not exhibit any toxic effect on the expression host. tFhuA is an extensive truncation of the Ferric hydroxamate uptake protein component A (FhuA).^18^ The primary gene, bn–tfhua, encoded Bn–tFhuA. This gene was developed using individual genes of Bn and tFhuA, bn and tfhua, respectively, and assembly PCR reactions.^19^ In addition, Bn–tFhuA included a short peptide adaptor (MGDRGPEFELGT),^57^ which was fused at the N terminus of Bn, a flexible hexapeptide tether ((GGS)2) between Bn and tFhuA, and KpnI sites at both 5′ and 3′ ends. The gene that encoded the barstar (Bs) protein ligand was subcloned into the pPR-IBA1 expression vector using BsaI restriction sites. This gene featured a double-alanine mutation, C40A/C82A.^58^ The genes that encoded weakly binding D39A Bs and moderately binding E76A Bs were created using inverse PCR protocols.^19,59^

### 4.2 Protein Expression and Purification

All genes were transformed using *E. coli* BL21 (DE3) cells. Protocols for protein expression and purification of Bn–tFhuA were previously reported.^17,60^ In the case of Bs, D39A Bs, and E76A Bs, transformed cells were grown in Luria–Bertani medium at 37 °C until OD600 reached ∼0.5. Then, the temperature was reduced to 20 °C. The cells were induced by adding IPTG when OD600 got a value of ∼0.7. The cell growth was conducted at 20 °C for another ∼18 h, then centrifuged at 4150g at 4 °C for 30 min. The pellet was resuspended in 150 mM KCl, 50 mM Tris-HCl, 5 mM EDTA, and pH 8.0. The cells were lysed using a model 110L microfluidizer (Microfluidics, Newton, MA) and centrifuged at 108,500g at 4 °C for 30 min. The supernatant was processed through ammonium sulfate precipitation and extensively dialyzed at 4 °C overnight against 20 mM Tris-HCl and pH 8.0. A Q-Sepharose ion exchange column (Bio-Rad, Hercules, CA) was used to purify the dialyzed supernatant. This purification process employed a linear salt gradient of 0–1 M KCl, 20 mM Tris-HCl, pH 8.0. Then, a refining purification step followed through the size-exclusion chromatography using a Superdex-75 column (GE Healthcare Life Sciences, Pittsburgh, PA). Purified protein samples were analyzed through SDS-PAGE and stored at −80 °C.

### 4.3 Refolding of Bn–tFhuA

Lyophilized Bn–tFhuA samples were solubilized in 200 mM KCl, 50 mM Tris-HCl, 8 M urea, and pH 8.0 to reach a concentration of ∼15 μM. Then, the protein samples were at room temperature for several hours before refolding. Detergent-mediated refolding of Bn–tFhuA was conducted by adding *n*-dodecyl-β-d-maltopyranoside (DDM) to a final concertation of 1.5% (w/v), which was followed by a slow dialysis against 200 mM KCl, 20 mM Tris-HCl, and pH 8.0, at 4 °C for at least 3 days. The refolded proteins were 20-fold diluted in 200 mM KCl, 20 mM Tris-HCl, 0.5% (w/v) DDM, and pH 8.0. Protein concentrations were assessed through their molar absorptivity at a wavelength of 280 nm.

### 4.4 Single-Molecule PPI Detection Using the Resistive-Pulse Technique with Planar Lipid Membranes

Single-channel electrical recordings were performed, as previously described.^35,61^ Lipid bilayers were prepared using 1,2-diphytanoyl-*sn*-glycero-phosphatidylcholine (Avanti Polar Lipids) across a ∼90 μm-diameter aperture in the Teflon partition separating the two symmetrical halves of the chamber. The electrolyte solution contained 300 mM KCl, 10 mM Tris-HCl, pH 8.0. Bn–tFhuA and protein ligands were added to the cis side of the chamber, which was at the ground. The final Bn–tFhuA concentration was between 0.3 and 1 ng/μL. The currents were recorded utilizing an Axopatch 200B patch-clamp amplifier (Axon Instruments, Foster City, CA). The transmembrane potential was −40 mV. The analog electrical signal was low-pass filtered at a frequency of 10 kHz using an 8-pole model 900 Bessel filter (Frequency Devices, Ottawa, IL). Then, the signal was digitized utilizing a low-noise acquisition system (model Digidata 1440A; Axon) and sampled at a rate of 50 kHz. pClamp 10.5 software package (Axon) was used for data acquisition. All single-channel electrophysiology measurements were performed at room temperature.

### 4.5 Statistical Analysis of Single-Molecule Events

ClampFit 10.7 (Axon) and Origin 8.5 (OriginLab, Northampton, MA) were utilized to prepare figures. The maximum likelihood method (MLM)^32^ and logarithm likelihood ratio (LLR)^33−35^ tests were used to fit event duration histograms. These methods were used to determine the number of statistically significant subpopulations best represented by the data. Digitized and sampled data were binned on a logarithmic time scale because of its superior resolution for widely separated time constants. Logarithmic two-exponential likelihood fits were constructed for analyzing ligand-captured and ligand-released duration histograms of competitive PPIs at various ligand concentrations. The fitting method in ClampFit (Axon) was a variable metric with a maximum likelihood estimation.

### 4.6 Determination of the Binding Event Probabilities, Fractional Occupancies, and Receptor Occupancies of Competitive PPIs

Let us consider a binary mixture containing two protein ligands, L_1_ and L_2_. The concentrations of these protein ligands are denoted by [L_1_] and [L_2_], respectively. They competitively interact against a single-tethered receptor R. Here, [L_1_] is kept constant, whereas [L_2_] is a variable concentration. The experimental value of the event probability of the L_2_-captured events, P_2_^Exp^ depends on the varying [L_2_] value, as follows
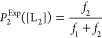
3here, *f*_1_ and *f*_2_ are the model-independent measured event frequencies of L_1_-captured and L_2_-captured events, respectively, in a competitive PPI experiment. They can be determined from individual peaks of capture duration histograms. A different way to determine this probability is to employ kinetic parameters determined from individual noncompetitive PPIs and assume ligand concentration-independent durations of capture events in competitive PPIs. This assumption is in accordance with our findings tabulated in the Supporting Information file. This way, we can determine the model-dependent event probability, P_2_^Mod^ at various [L_2_] values
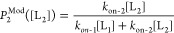
4*k*_on-1_ and *k*_on-2_ denote the association rate constants of the L_1_- and L_2_-captured events, respectively. Hence, *P*_1_^Mod^ can be calculated using *k*_on-1_ and *k*_on-2_ from individual noncompetitive PPI experiments (e.g., with either varying [L_1_] or varying [L_2_]). This calculation provides opportunities for comparing model-independent (experimental; *P*_2_^Exp^) with model-dependent values of *P*_2_ (*P*_2_^Mod^). Yet, a more relevant measure of the competitive PPIs for macroscopic determinations is the fractional occupancy, *F*, which is the total time of a specific L_2_-occupied Bn site normalized to the total time of ligand-occupied Bs site. For L_2_, the experimental fractional occupancy is given by the following expression
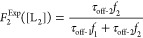
5here, τ_off-1_ and τ_off-2_ are the dissociation rate constants of the L_1_- and L_2_-captured events, respectively. Again, the experimental value of *F*_2_, *F*_2_^Exp^, can be calculated as a function depending on the event frequencies, *f*_1_ and *f*_2_, as well the dissociation rate constants, τ_off-1_ and τ_off-2_. Only *f*_1_ and *f*_2_ depend on [L_2_]. The model-dependent *F*_2_, *F*_2_^Mod^, can be obtained using the corresponding *K*_D_ constants, *K*_D-1_ and *K*_D-2_, and the ligand concentrations of both protein ligands, [L_1_] and [L_2_], as follows
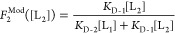
6

Finally, the receptor occupancy of the Bs binding site, *O*([L_2_]), is defined as the total time of ligand-occupied events normalized to the total recording time. The model-dependent occupancy, *O*^Mod^([L_2_]), is given by the following expression (Supporting Information, Methods)
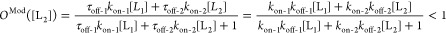
7

The model-dependent occupancies are determined using kinetic parameters acquired from noncompetition (e.g., single-ligand experiments) and experimental protein concentrations. For the experimental receptor occupancy, the following formula was utilized
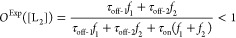
8where dwell times and frequencies are directly obtained using standard semilogarithmic histograms at different concentrations of the competing protein ligand, [L_2_].

### 4.7 Two-Ligand, One-Receptor Queuing Model of Competitive PPIs

To analyze pore-protein complex competitive binding, we employ the queuing theory approach,^62,63^ a probabilistic analysis of waiting lines. Queuing models have been applied to a range of biological processes at a molecular level,^64−66^ which include multisite enzyme kinetics,^67,68^ gene expression,^69,70^ and metabolic networks.^71^ Here, ligands randomly arrive at the nanopore, transiently bind, and are released, allowing another ligand to engage with the nanopore. We model the arrival of a ligand as a Poisson process with rate λ, while the rate of release of the ligand that is bound is given with rate of service μ. The probability that a pore is bound at any moment in time is given by blocking probability (Erlang B formula)^72^ that we apply here for the 1-pore system
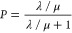
9

At a steady state, the rate of arrivals is given by the concentration of ligands and pore-ligand binding rate
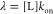
10while the rate constant of service, μ, is given by the dissociation rate constant
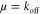
11

Hence, the probability that the pore is occupied by a single ligand is the following
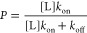
12here [L], *k*_on_, and *k*_off_ are experimentally determined values for single-ligand experiments. This quantity accurately predicts the receptor occupancy measured in experiments with a single ligand type (Figure 2f). Then, we model competitive binding in binary mixtures as a process balanced by pore occupancies.
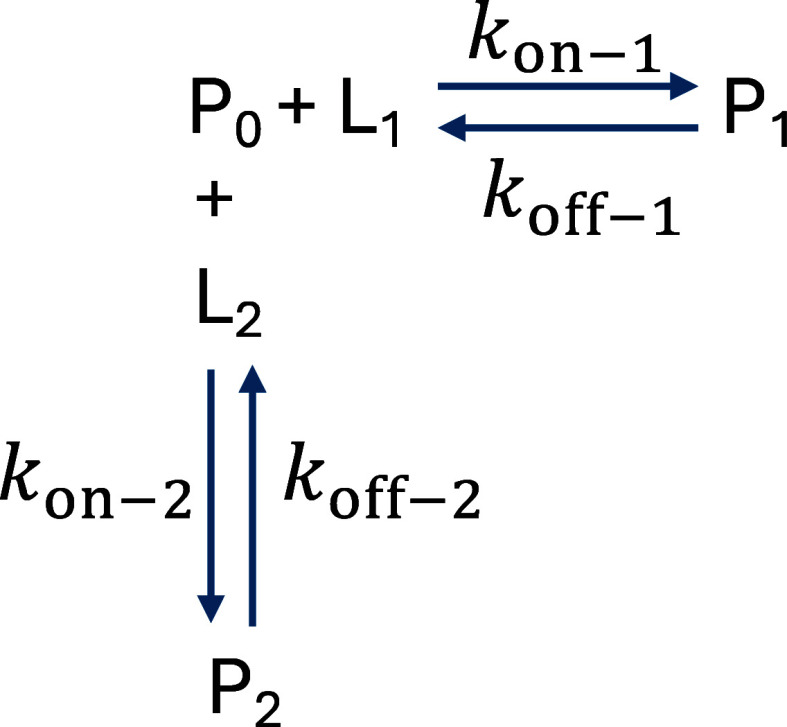


Here, L_*i*_ denotes protein ligand “*i*”, *P*_0_ is the probability that the pore is unoccupied, and *P*_*i*_ is the probability that the pore is occupied by ligand *i* given by eq 9 for each ligand. The forward and reverse rates for ligand “*i*” are given by λ_*i*_ and μ_*i*_, respectively. The system is balanced when the flux to the unoccupied state equals the flux to occupied states *P*_*i*_
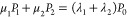
13

We solve for *P*_0_ by setting.
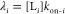
14
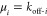
15to obtain
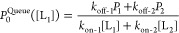
16where
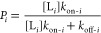
17

Hence, the receptor occupancy is given by the following formula
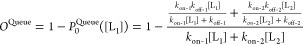
18which always exhibits a minimum. If the first ligand concentration, [L_1_], is constant, and the second ligand concentration, [L_2_], is variable, then the condition for the minimum receptor occupancy is the following
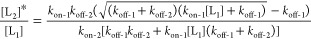
19

Here, [L_2_]* is the critical competing ligand concentration at which the receptor occupancy reaches a minimum value. Under these conditions, the minimum value of the receptor occupancy is given by
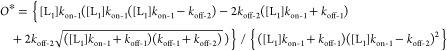
20

### 4.8 Molecular Graphics

A molecular graphic was prepared utilizing the PyMOL Molecular Graphics System (Version 2.4.0; Schrödinger, LLC). In this article, we used entries 1BY3.pdb (FhuA),^18^ 1BRS.pdb (Bn–Bs),^22^ and 1BTA.pdb (Bs).^73^

## Supporting Information Available

Calculation of the model-dependent value of the receptor occupancy, Scatter plots of ligand-released and ligand-captured events, tables of the mean values of the current amplitudes of ligand-captured and ligand-released events, standard event histograms of ligand-released and ligand-captured durations in a linear representation, standard all-point current amplitude histograms of ligand-released and ligand-captured substates, tables of time and rate constants of the binding events produced by individual protein ligands, supplementary single-channel electrical traces, and amplitude histograms produced by individual protein ligands and binary mixtures of competing protein ligands, tables of time and rate constants of the binding events produced by binary mixtures of competing protein ligands, tables of event probability, fractional occupancy, and receptor occupancy in the presence of a binary mixture of competing protein ligands with strong and moderate binding affinities, analytical modeling of the fractional occupancies of the competing protein ligands for various binary mixtures of ligands, tables of event probability, fractional occupancy, and receptor occupancy in the presence of a binary mixture of competing protein ligands with strong and weak binding affinities (PDF)
