# Supplementary material for: Single-Molecule Observation of Competitive Protein–Protein Interactions Utilizing a Nanopore
Source: ACS Nano. 2024 Dec 24;19(1):1103–15. doi: 10.1021/acsnano.4c13072 (PMC11752528; doi:10.1021/acsnano.4c13072)
Supplement: Supplementary file 1 — nn4c13072_si_001.pdf [file nn4c13072_si_001.pdf]

SUPPLEMENTARY INFORMATION FILE

Single-Molecule Observation of Competitive Protein-Protein Interactions Utilizing a Nanopore

Jiaxin Sun[1], Antun Skanata[1,2], and Liviu Movileanu[1,2,3,4&]

[1]*Department of Physics, Syracuse University, 201 Physics Building, Syracuse, New York 13244-1130, USA*

[2]*The BioInspired Institute, Syracuse University, Syracuse, New York 13244, USA*

[3]*Department of Biomedical and Chemical Engineering, Syracuse University, 329 Link Hall, Syracuse, New York 13244, USA*

[4]*Department of Biology, Syracuse University, 114 Life Sciences Complex, Syracuse, New York 13244, USA*

Running title: Competitive protein-protein interactions utilizing a nanopore

[&]The corresponding author:
E-mail: lmovilea@syr.edu

SUPPLEMENTAL METHODS

Calculation of the model-dependent value of the occupancy. Here, we write the expressions for individual ligand-released durations, $\tau_{\text{on}-1}$ and $\tau_{\text{on}-2}$, in a binary mixture of ligands:[1]

$$\tau_{\text{on}-1} = \frac{\tau_{\text{on}}}{P_1} \quad \text{(S1)}$$

$$\tau_{\text{on}-2} = \frac{\tau_{\text{on}}}{P_2} \quad \text{(S2)}$$

where τ_{on} is the experimentally determined ligand-released duration, which is inferred through standard event histograms (e.g., **Figure 3b** and **Figure 4b**).

$$1 = P_1 + P_2 = \frac{\tau_{\text{on}}}{\tau_{\text{on}-1}} + \frac{\tau_{\text{on}}}{\tau_{\text{on}-2}} \quad \text{(S3)}$$

where P_1 and P_2 are the event probabilities of the two protein ligands from the mixture. Hence,

$$\frac{1}{\tau_{\text{on}}} = \frac{1}{\tau_{\text{on}-1}} + \frac{1}{\tau_{\text{on}-2}} \quad \text{(S4)}$$

In this model, we assume that bindings of the protein ligands L_1 and L_2 to the receptor R independently occur with respect to each other (e.g., they do not wait for each other).

Hence

$$k_{\text{on}-1} = \frac{1}{[L_1]\tau_{\text{on}-1}} \quad \text{(S5)}$$

$$k_{\text{on}-2} = \frac{1}{[L_1]\tau_{\text{on}-2}} \quad \text{(S6)}$$

where $[L_1]$ and $[L_2]$ are the effective concentrations of the protein ligands in the mixture. Here, $k_{\text{on}-1}$ and $k_{\text{on}-2}$ are the association rate constants of each protein ligand, as determined from non-competition binding assays. Therefore, by combining the Eqns. (S4)-(S6), we obtain

$$\tau_{\text{on}} = \frac{1}{k_{\text{on}-1}[L_1] + k_{\text{on}-2}[L_2]} \quad \text{(S7)}$$

Let's denote by N_1 and N_2 the number of capture events made by the protein ligands L_1 and L_2, respectively. Then, the total recording time, T_r, is given by the following expression:

$$T_r = \tau_{\text{off}-1}N_1 + \tau_{\text{off}-2}N_2 + \tau_{\text{on}}(N_1 + N_2)$$

where $\tau_{\text{off}-1}$and $\tau_{\text{off}-2}$ are the mean capture durations obtained from semilogarithmic time histograms (e.g., **Figure 3c** and **Figure 4c**).

The model-dependent pore occupancy is given by:

$$O^{\text{Mod}}[L_1] = \frac{\tau_{\text{off}-1}N_1 + \tau_{\text{off}-2}N_2}{\tau_{\text{off}-1}N_1 + \tau_{\text{off}-2}N_2 + \tau_{\text{on}}(N_1 + N_2)} = \frac{\tau_{\text{off}-1}P_1 + \tau_{\text{off}-2}P_2}{\tau_{\text{off}-1}P_1 + \tau_{\text{off}-2}P_2 + \tau_{\text{on}}} \quad \text{(S8)}$$

where

$$P_1^{\text{Mod}}([L_1]) = \frac{k_{\text{on}-1}[L_1]}{k_{\text{on}-1}[L_1] + k_{\text{on}-2}[L_2]} \quad \text{(S9)}$$

and

$$P_2^{\text{Mod}}([L_1]) = \frac{k_{on-2}[L_1]}{k_{\text{on}-1}[L_1] + k_{\text{on}-2}[L_2]} \quad \text{(S10)}$$

Introducing the expressions (**S7**), (**S9**), and (**S10**) into (**S8**), the model-dependent occupancy is the following formula:

$$O^{\text{Mod}}[L_1] = \frac{\tau_{\text{off}-1}k_{\text{on}-1}[L_1] + \tau_{\text{off}-2}k_{\text{on}-2}[L_2]}{\tau_{\text{off}-1}k_{\text{on}-1}[L_1] + \tau_{\text{off}-2}k_{\text{on}-2}[L_2] + 1} \quad \text{(S11)}$$

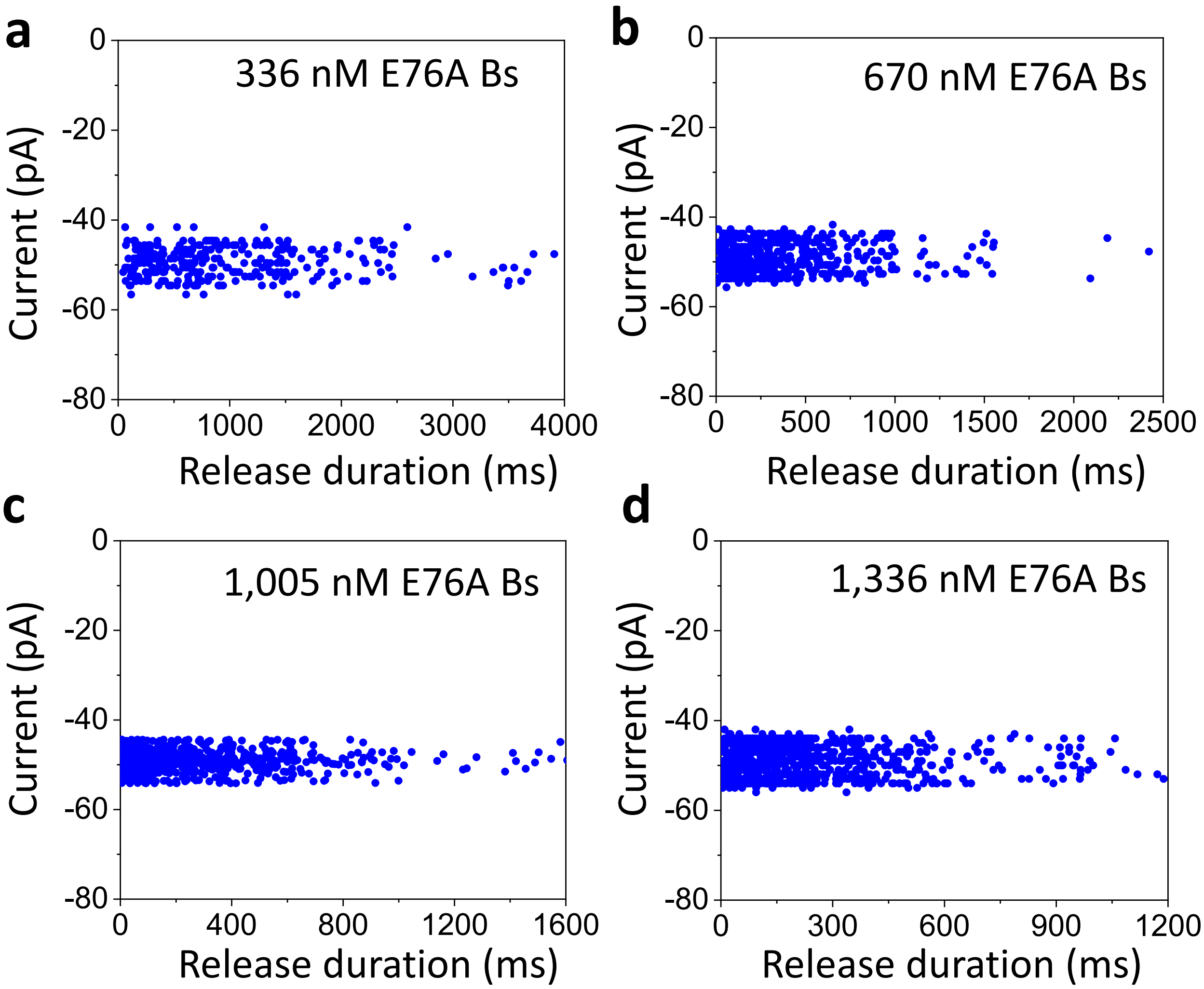

Supplementary Figure S1. Scatter plots of E76A Bs-released current blockades versus E76A Bs-released durations from a single representative single-channel electrical trace. Scatter plots are illustrated at various [E76A Bs] values. **(a)** 336 nM E76A Bs. **(b)** 670 nM E76A Bs. **(c)** 1,005 nM E76A Bs. **(d)** 1,336 nM E76A Bs.

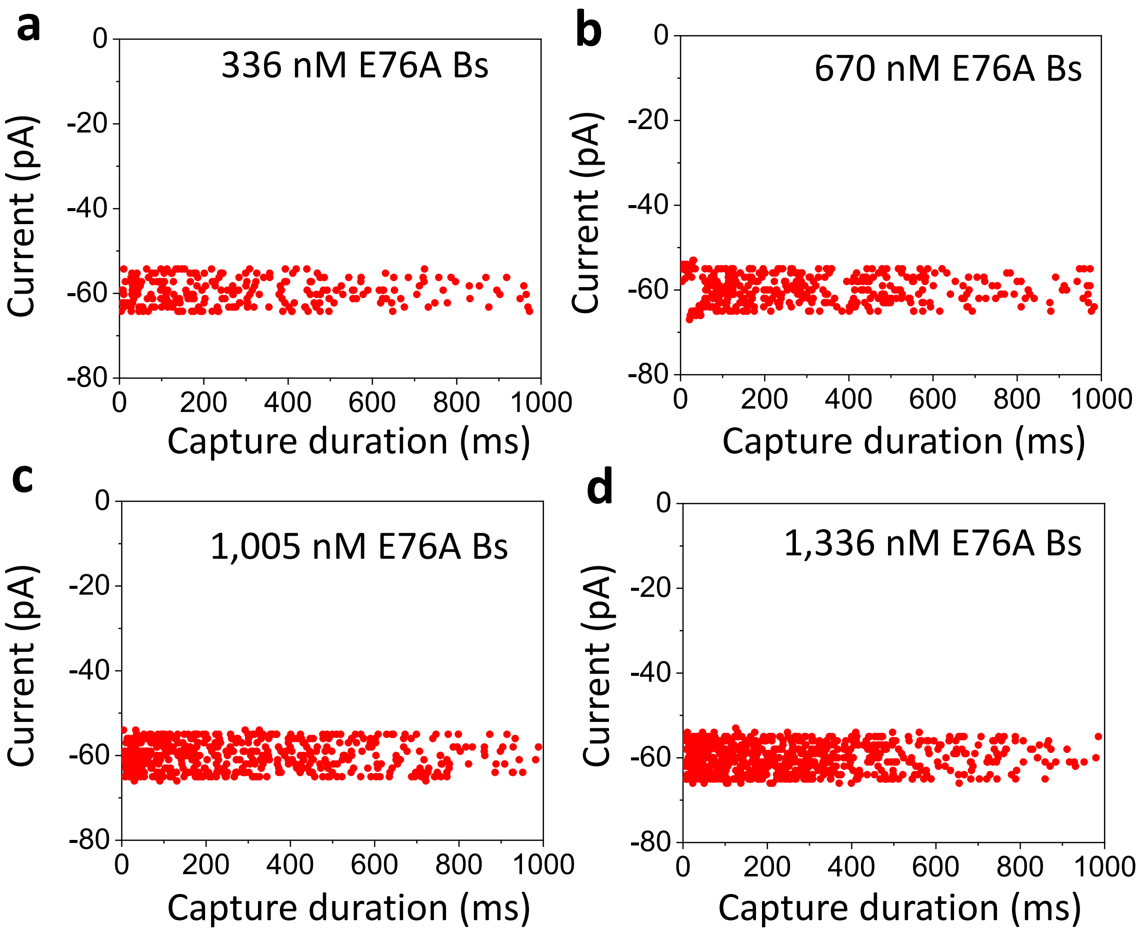

Supplementary Figure S2. Scatter plots of E76A Bs-produced current blockades versus E76A Bs capture durations from a single-channel electrical trace. Scatter plots are illustrated at various [E76A Bs] values. **(a)** 336 nM E76A Bs. **(b)** 670 nM E76A Bs. **(c)** 1005 nM E76A Bs. **(d)** 1336 nM E76A Bs.

Supplementary Table S1. The mean values of the current amplitudes of the substates O_{on} and O_{off} for the Bn-E76A Bs interactions from Fig. 2b and Supplementary Fig. S3. The other recording conditions are indicated in **Experimental section**. Values are mean ± s.e.m. from a representative single-channel electrical trace.

**[E76A Bs] (nM)**	**O_{on} (pA)**	**O_{off} (pA)**
0	48.9 ± 0.1	NA
336	48.6 ± 0.1	59.3 ± 0.1
670	48.6 ± 0.1	59.4 ± 0.1
1,005	48.2 ± 0.3	58.8 ± 0.1
1,336	49.6 ± 1.3	59.0 ± 0.1
Mean*	48.8 ± 0.5	59.1 ± 0.3

NA stands for not applicable.

*This line indicates data directly averaged from data collected at different [E76A Bs] values.

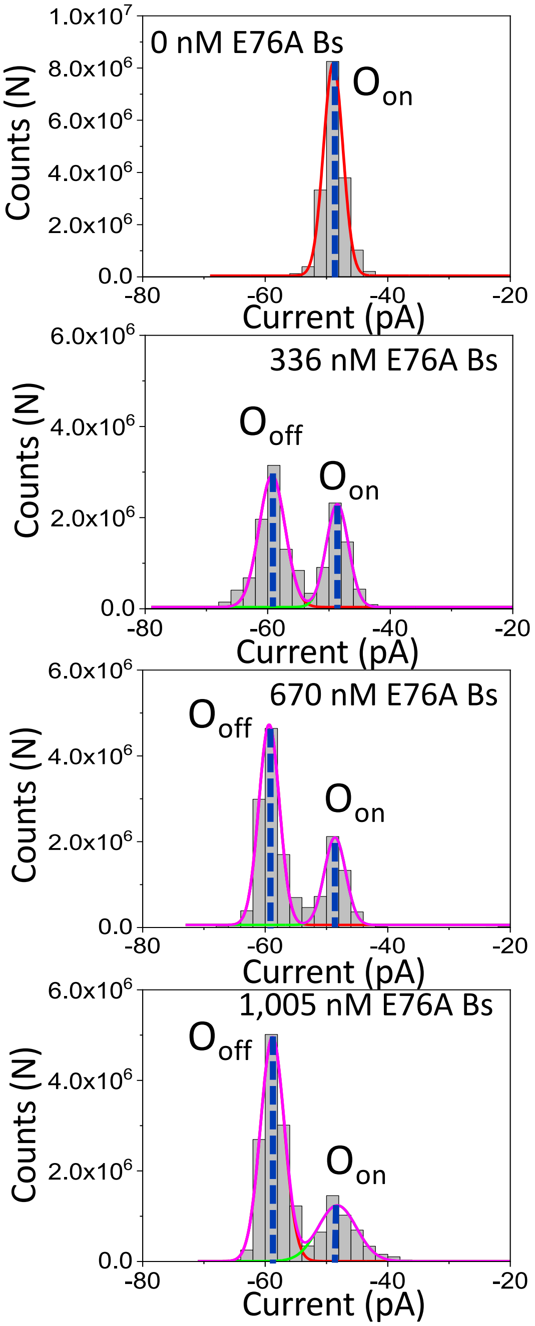

<u>Figure S3.</u> All-point current amplitude histogramns of Bn-tFhuA in the presence of E76A Bs. These histograms are provided for 0 nM, 336 nM, 670 nM, and 1,005 nM E76A Bs from the top to bottom, respectively. The Gaussian peak maxima indicate the current amplitudes corresponding to the "off" (Bs-captured event, O_{off}) and "on" (Bs-released event, O_{on}) substates. On the top panel, the red line corresponds to the fit of the O_{on} substate. On the other panels, the green and red lines correspond to the fits of the O_{on} and O_{off} substates, respectively. The magenta line is the cumulative fit.

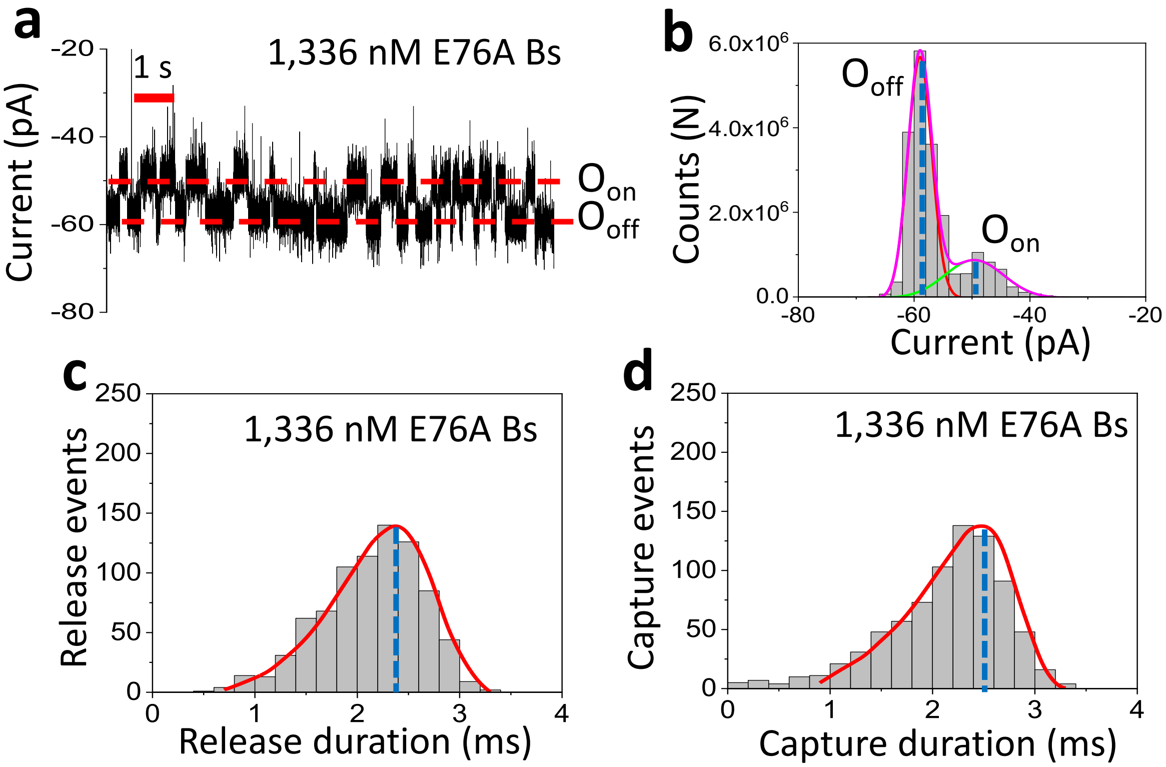

<u>Supplementary Figure S4.</u> **Determination of the single-channel electrical signature of Bn-tFhuA when 1,336 nM E76A Bs was added to the *cis* side of the chamber. (a)** A representative single-channel electrical trace was low-pass filtered at 1 kHz using an 8-pole Bessel filter. The other recording conditions are indicated in **Experimental section**. (**b**) The corresponding all-point current amplitude histogram. Maxima of the peaks of the current amplitude represent the substates "off" (E76A Bs-captured event, O_{off}) and "on" (Bs-released event, O_{on}). The following values are provided as mean ± s.e.m.: O_{on} and O_{off} were -49.6 ± 1.3 pA and -59.0 ± 0.1 pA, respectively. **(c)** A standard event histogram of E76A Bs-released durations. The τ_{on} release duration (mean ± s.e.m.) was 245 ± 6 ms (N = 807 events). **(d)** A standard event histogram of E76A Bs-captured durations. The τ_{off} capture duration (mean ± s.e.m.) was 323 ± 11 ms (N = 792 events). The maximum likelihood method (MLM)[2] and logarithm likelihood ratio (LLR)[3-5] tests were used to fit event duration histograms. These methods were used to determine the number of statistically significant subpopulations best represented by the data.

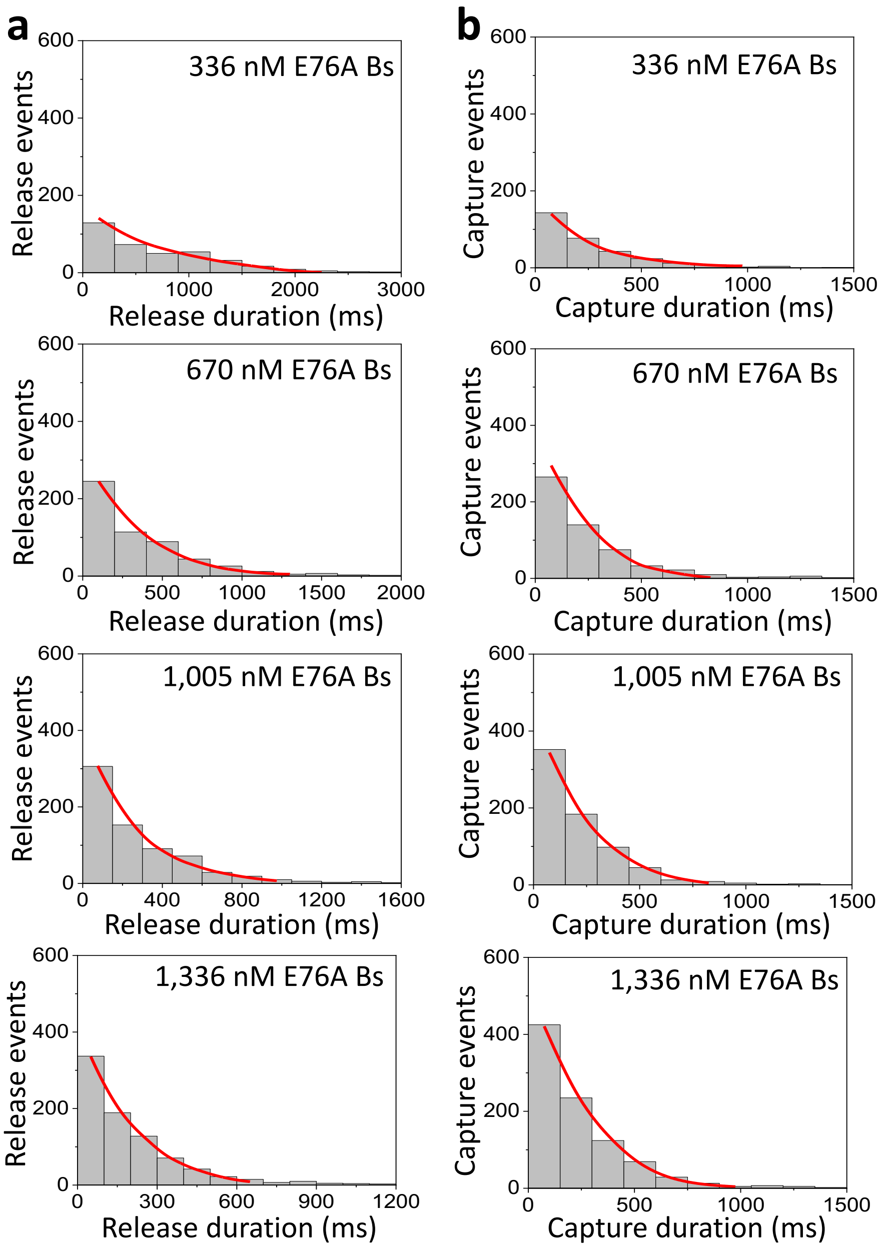

Supplementary Figure S5. Event histograms of E76A Bs-produced current blockades at various [E76A Bs] values. (a) Event histograms of E76A Bs-released durations. The τ_{on} release durations (mean ± s.e.m.) from these histogram fits were 1,015 ± 44 ms (the number of events: N = 322), 421 ± 35 ms (N = 506), 309 ± 31 ms (N = 657), and 246 ± 17 ms (N = 807 events) at [E76A Bs] values of 336 nM, 670 nM, 1005 nM, and 1,336 nM, respectively. **(b)** Event histograms of E76A Bs-captured durations. The τ_{off} capture durations (mean ± s.e.m.) from these histogram fits were 302 ± 19 ms (number of events: N = 316), 296 ± 33 ms (N = 497), 304 ± 9 ms (N = 646), and 327 ± 23 ms (N = 792 events) at [E76A Bs] values of 336 nM, 670 nM, 1,005 nM, and 1,336 nM, respectively. The other recording conditions are indicated in the **Experimental section**. The maximum likelihood method (MLM)[2] and logarithm likelihood ratio (LLR)[3-5] tests were used to fit event duration histograms. These methods were used to determine the number of statistically significant subpopulations best represented by the data.

Supplementary Table S2. The mean values of the τ_{on} and τ_{off} time constants, as well as the k_{on} and k_{off} kinetic rate constants of the Bn-E76A Bs interaction from a single representative experiment. Data are extracted from **Fig. 2c**, **Fig. 2d**, and **Supplementary Fig. S3cd**. Values are mean ± s.e.m. from a representative single-channel electrical trace. The maximum likelihood method (MLM)[2] and logarithm likelihood ratio (LLR)[3-5] tests were used to fit event duration histograms. These methods were used to determine the number of statistically significant subpopulations best represented by the data. The other recording conditions are indicated in **Experimental Section**.

**[E76A Bs] (nM)**	**τ_{on} (ms)**	**τ_{off} (ms)**	**k_{on} (10^7 $M^{-1}s^{-1}$)**	**k_{off} (s^{-1})**	**K_D (μM)**
336	1,013 ± 39	313 ± 12	0.29 ± 0.01	3.2 ± 0.1	1.1 ± 0.1
670	416 ± 36	298 ± 17	0.36 ± 0.03	3.4 ± 0.2	0.9 ± 0.1
1,005	309 ± 38	307 ± 14	0.32 ± 0.04	3.3 ± 0.2	1.0 ± 0.1
1,336	245 ± 6	323 ± 11	0.31 ± 0.01	3.1 ± 0.1	1.0 ± 0.1
Mean*	NA	310 ± 10	0.32 ± 0.02	3.3 ± 0.1	1.0 ± 0.1

NA stands for not applicable.
*This line indicates data directly averaged from data collected at different [E76A Bs] values.

Supplementary Table S3. The mean values of the τ_{on} and τ_{off} time constants, as well as the k_{on} and k_{off} kinetic rate constants for the Bn-E76A Bs interactions. The maximum likelihood method (MLM)[2] and logarithm likelihood ratio (LLR)[3-5] tests were used to fit event duration histograms. These methods were used to determine the number of statistically significant subpopulations best represented by the data. Values are mean ± s.d. using $n = 3$ independently conducted experiments. The other recording conditions are indicated in the **Experimental Section**.

**[E76A Bs] (nM)**	**τ_{on} (ms)**	**τ_{off} (ms)**	**k_{on} (10^7 $M^{-1}s^{-1}$)**	**k_{off} (s^{-1})**	**K_D (μM)**
Fit*	NA	NA	0.32 ± 0.05	3.5 ± 0.1	1.1 ± 0.1
336	1,026 ± 77	284 ± 30	0.29 ± 0.02	3.6 ± 0.4	1.2 ± 0.2
670	439 ± 21	285 ± 13	0.34 ± 0.02	3.5 ± 0.2	1.0 ± 0.1
1,005	321 ± 23	281 ± 27	0.31 ± 0.02	3.6 ± 0.4	1.2 ± 0.2
1,336	234 ± 14	291 ± 29	0.32 ± 0.02	3.5 ± 0.3	1.1 ± 0.1
Mean**	NA	285 ± 4	0.32 ± 0.02	3.6 ± 0.1	1.1 ± 0.1

*This line provides data directly acquired from the linear-regression fits in **Figs. 2ef**. Data are reported as mean ± s.e.m.
**This line indicates data directly averaged from data collected at different [E76A Bs] values.
NA stands for not applicable.

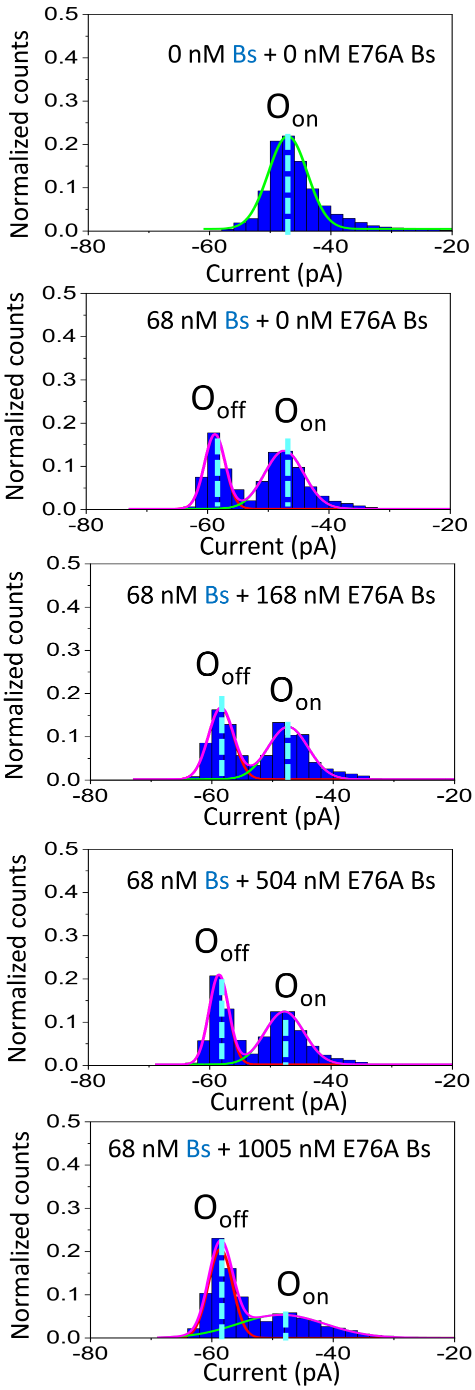

Supplementary Table S6. Normalized all-point current amplitude histograms are provided for various binary mixtures containing 68 nM Bs and titratable [E76A Bs]. Gaussian two-peak fits were generated. Maxima of the peaks of the current amplitude represent the substates "off" (ligand-captured event, O_{off}, *left*) and "on" (ligand-released event, O_{on}, *right*). On the top panel, the green line corresponds to the fit of the O_{on} substate. On the other panels, the green and red lines correspond to the fits of the O_{on} and O_{off} substates, respectively. The magenta line is the cumulative fit.

Supplementary Table S4. **Capture (τ_{off}) and release (τ_{on}) durations of the binding events in a binary mixture of protein ligands with strong and moderate affinity.** 68 nM Bs was added to the *cis* side of the chamber. The binary mixture also encompassed a varying [E76A Bs]. k_{off} is the dissociation rate constant. The maximum likelihood method (MLM)[2] and logarithm likelihood ratio (LLR)[3-5] tests were used to fit event duration histograms. These methods were used to determine the number of statistically significant subpopulations best represented by the data. The other recording conditions are indicated in the **Experimental section**. Values are mean ± s.d. from n = 3 independently reconstituted nanopores.

[E76A Bs] (nM)	$\tau_{off\text{-}Bs}$ (ms)	$\tau_{off\text{-}E76A\ Bs}$ (ms)	τ_{on} (ms)	$k_{off\text{-}Bs}$ (s^{-1})	$k_{off\text{-}E76A\ Bs}$ (s^{-1})
0	1,057 ± 85	NA	995 ± 59	0.95 ± 0.07	NA
168	929 ± 35	267 ± 13	945 ± 58	1.08 ± 0.04	3.75 ± 0.19
336	932 ± 37	273 ± 14	889 ± 63	1.07 ± 0.04	3.67 ± 0.19
504	935 ± 41	278 ± 11	576 ± 24	1.07 ± 0.05	3.60 ± 0.14
672	926 ± 30	271 ± 15	415 ± 27	1.08 ± 0.04	3.70 ± 0.21
1,005	943 ± 40	279 ± 17	304 ± 23	1.06 ± 0.05	3.59 ± 0.22
1,586	914 ± 30	265 ± 5	207 ± 17	1.09 ± 0.04	3.78 ± 0.08
Mean*	930 ± 10	272 ± 6	NA	1.08 ± 0.01	3.68 ± 0.08

NA stands for not applicable.
*This line indicates data directly averaged from data collected at different [E76A Bs] values.

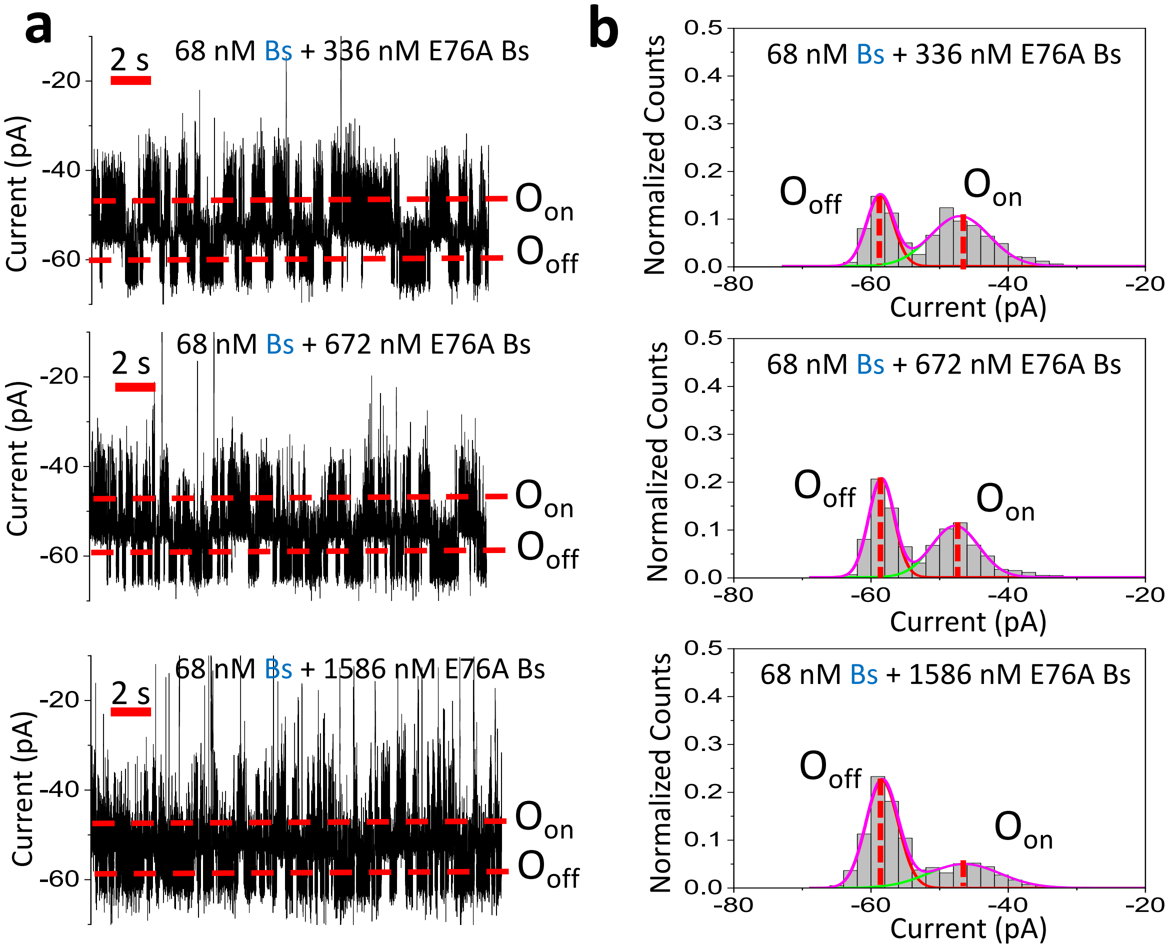

<u>Supplementary Figure S7.</u> Competitive PPIs with strongly and moderately binding interactions. (a) Representative single-channel electrical traces were filtered at 1 kHz using a low-pass 8-pole Bessel filter for binary mixtures of strongly and moderately binding protein ligands. The protein binary mixture included 68 nM Bs and various [E76A Bs] values added to the *cis* side of the chamber. The O_{on} and O_{off} levels represent the ligand-released and ligand-captured substates, respectively. The applied transmembrane potential was -40 mV. These single-channel electrical traces represent a subset of $n = 3$ distinct nanopores. The other recording conditions are indicated in the **Experimental section**. **(b)** Corresponding normalized all-point current amplitude histograms are provided for binary mixtures of 68 nM Bs and various [E76A Bs] values. Multiple-peak fittings are generated. Maxima of the peaks of the current amplitude represent the substates "off" (ligand-captured event, O_{off}, *left*) and "on" (ligand-released event, O_{on}, *right*).

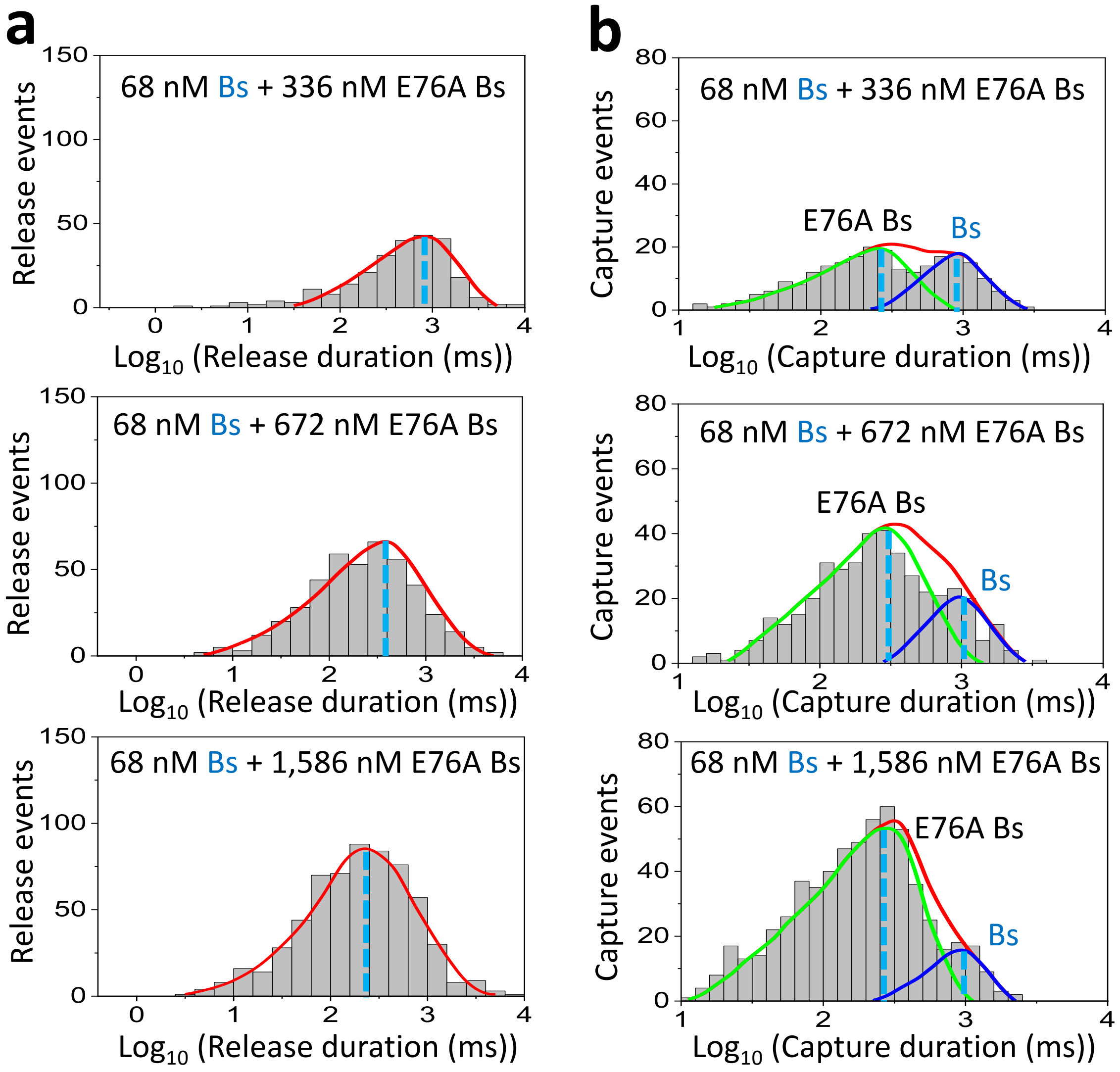

<u>**Supplementary Figure S8.**</u> **Semilogarithmic event duration histograms resulting from competitive PPIs with strongly and moderately binding interactions.** (**a**) (Representative semilogarithmic duration histograms of ligand-released events at various [E76A Bs] values. The red curves represent the cumulative fits for the ligand-released durations. The τ_{on} release durations (mean ± s.e.m.) from these histograms were 828 ± 38 ms (the number of events: N = 251), 397 ± 53 ms (N = 434), and 226 ± 29 ms (n = 612) at 336 nM E76A Bs, 672 nM E76A Bs, and 1,586 nM E76A Bs, respectively. (**b**) Representative semilogarithmic duration histograms of ligand-captured events at various [E76A Bs] values. The τ_{off} capture durations (mean ± s.e.m.) from these histograms were 257 ± 38 ms and 891 ± 63 ms (the number of events: N = 244), 288 ± 36 ms and 954 ± 51 ms (N = 427), and 269 ± 35 ms and 924 ± 38 ms (N = 608) at 336 nM E76A Bs, 672 nM E76A Bs, and 1,586 nM E76A Bs, respectively. The other recording conditions are indicated in **Experimental section**. The maximum likelihood method (MLM)[2] and logarithm likelihood ratio (LLR)[3-5] tests were used to fit event duration histograms. These methods were used to determine the number of statistically significant subpopulations best represented by the data.

<u>Supplementary Table S5.</u> The mean values of the current amplitudes of the substates O_{on} and O_{off} from Fig. 3b. The protein binary mixture included 68 nM Bs and various [E76A Bs] values added to the *cis* side of the chamber. Without protein ligands, the O_{on} state corresponded to −47.1 ± 0.1 pA. The other recording conditions are indicated in **Experimental section**. Values are mean ± s.e.m. from a representative single-channel electrical trace**.**

[E76A Bs] (nM)	O_{on} (pA)	O_{off} (pA)
0	−47.3 ± 0.1	−58.8 ± 0.1
168	−47.4 ± 0.2	−58.4 ± 0.1
504	−47.9 ± 0.2	−58.5 ± 0.1
1,005	−48.9 ± 1.9	−58.3 ± 0.1
Mean*	−47.9 ± 0.7	−58.5 ± 0.2

*This line indicates data directly averaged from data collected at different [E76A Bs] values.

<u>Supplementary Table S6.</u> Release durations of individual binding events in a binary mixture of protein ligands with strong and moderate affinity. 68 nM Bs was added to the *cis* side of the chamber. The binary mixture of ligands encompassed a varying [E76A Bs]. $\tau_{on\text{-}Bs} = \tau_{on} / P_{Bs}$, where P_{Bs} is the event probability of the Bs-captured events (**Eqn. (1)**). $\tau_{on\text{-}E76A\ Bs} = \tau_{on} / P_{E76A\ Bs}$, where $P_{E76A\ Bs}$ is the event probability of the E76A Bs-captured events (**Eqn. (1)**). Here, τ_{on} are mean values of the single-exponential distributions of ligand-released duration histograms (**Supplementary Table S4**). k_{on} and K_D are the rate constant of association and the equilibrium dissociation constant, respectively. The other recording conditions are indicated in the **Experimental section**. The maximum likelihood method (MLM)[2] and logarithm likelihood ratio (LLR)[3-5] tests were used to fit event duration histograms. These methods were used to determine the number of statistically significant subpopulations best represented by the data. Values in the table are mean ± s.d. from n = 3 independently reconstituted nanopores.

[E76A Bs] (nM)	$\tau_{on\text{-}Bs}$ (ms)	$\tau_{on\text{-}E76A\ Bs}$ (ms)	$k_{on\text{-}Bs}$ (10^7 $M^{-1}s^{-1}$)	$k_{on\text{-}E76A\ Bs}$ (10^7 $M^{-1}s^{-1}$)	$K_{D\text{-}Bs}$ (nM)	$K_{D\text{-}E76A\ Bs}$ (μM)
0	995 ± 59	NA	1.48 ± 0.09	NA	64 ± 9	NA
168	1,626 ± 169	2,266 ± 37	0.91 ± 0.10	0.26 ± 0.01	119 ± 13	1.4 ± 0.1
336	2,135 ± 188	1,538 ± 211	0.69 ± 0.06	0.20 ± 0.02	156 ± 10	1.9 ± 0.2
504	2,149 ± 157	788 ± 35	0.69 ± 0.05	0.25 ± 0.01	156 ± 13	1.4 ± 0.1
672	1,861 ± 338	536 ± 19	0.81 ± 0.14	0.28 ± 0.01	137 ± 26	1.3 ± 0.1
1,005	2,007 ± 803	371 ± 59	0.83 ± 0.37	0.27 ± 0.04	145 ± 59	1.3 ± 0.2
1,586	1,789 ± 258	234 ± 18	0.83 ± 0.13	0.27 ± 0.02	133 ± 16	1.4 ± 0.1
Mean*	1,928 ± 206	NA	0.79 ± 0.09	0.26 ± 0.03	141 ± 14	1.5 ± 0.2

NA stands for not applicable.
*This line indicates data directly averaged from data collected at different [E76A Bs] values.

<u>Supplementary Table S7.</u> The probability (*P*), fractional occupancy (*F*), and occupancy (*O*) of E76A Bs-captured events in the presence of a binary mixture of protein ligands with strong and moderate affinity. 68 nM Bs was added to the *cis* side of the chamber. The binary mixture of ligands also encompassed various [E76A Bs] values. The other recording conditions are indicated in the **Experimental section**. Values are mean ± s.e.m. from a representative single-channel electrical trace**.**

[E76A Bs] (nM)	P_{Bs}	$P_{E76A\ Bs}$	F_{Bs}	$F_{E76A\ Bs}$	O^{Mod}	O^{Exp}
0	1	0	1	0	0.507	0.513
168	0.610 ± 0.013	0.390 ± 0.013	0.835 ± 0.024	0.165 ± 0.024	0.540	0.431
336	0.414 ± 0.047	0.586 ± 0.047	0.710 ± 0.039	0.290 ± 0.039	0.570	0.385
504	0.282 ± 0.032	0.718 ± 0.032	0.571 ± 0.037	0.429 ± 0.037	0.596	0.454
672	0.252 ± 0.014	0.748 ± 0.014	0.527 ± 0.025	0.473 ± 0.025	0.619	0.534
1,005	0.113 ± 0.010	0.887 ± 0.010	0.295 ± 0.016	0.705 ± 0.016	0.657	0.538
1,586	0.113 ± 0.009	0.887 ± 0.009	0.304 ± 0.012	0.696 ± 0.012	0.708	0.603

<u>Supplementary Table S8.</u> The probability (*P*), fractional occupancy (*F*), and true occupancy (*O*) of E76A Bs-captured events in the presence of a binary mixture of protein ligands with strong and moderate affinity. 68 nM Bs was added to the *cis* side of the chamber. The binary mixture also encompassed a varying [E76A Bs]. The other recording conditions are indicated in the **Experimental section**. Values in the table are mean ± s.d. from $n = 3$ independently reconstituted nanopores.

[E76A Bs] (nM)	P_{Bs}	$P_{E76A\ Bs}$	F_{Bs}	$F_{E76A\ Bs}$	O^{Mod}	O^{Exp}
0	1	0	1	0	0.507	0.515 ± 0.033
168	0.583 ± 0.026	0.417 ± 0.026	0.829 ± 0.011	0.171 ± 0.011	0.540	0.409 ± 0.023
336	0.419 ± 0.044	0.581 ± 0.044	0.709 ± 0.034	0.291 ± 0.034	0.570	0.382 ± 0.004
504	0.268 ± 0.015	0.732 ± 0.015	0.552 ± 0.019	0.448 ± 0.019	0.596	0.441 ± 0.018
672	0.226 ± 0.026	0.774 ± 0.026	0.499 ± 0.027	0.501 ± 0.027	0.619	0.502 ± 0.033
1,005	0.173 ± 0.085	0.827 ± 0.085	0.401 ± 0.138	0.599 ± 0.138	0.657	0.563 ± 0.030
1,586	0.117 ± 0.014	0.883 ± 0.014	0.313 ± 0.020	0.687 ± 0.020	0.708	0.622 ± 0.017

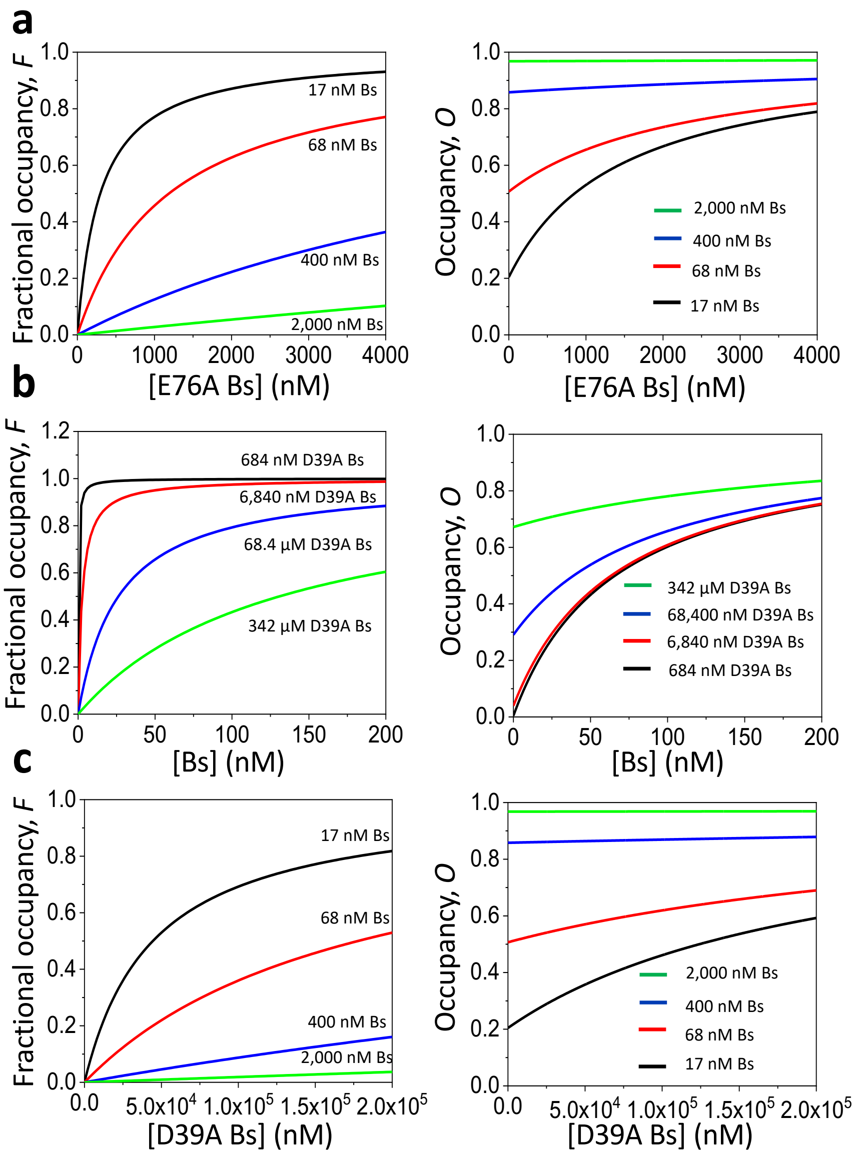

Supplementary Figure S9. Analytical modeling of the fractional occupancies, *F*, of the competitor proteins for various binary mixtures of ligands. (a) Simulations of the dependence of the probability of E76A Bs-captured events (*left panel*) and the dependence of the fractional occupancy of E76A Bs-captured events (*right panel*) on the [E76A Bs] value at various constant [Bs] values. The model data are based on the k_{on} and K_D generated from the individual Bn-Bs and Bn-E76A Bs binding assays ($k_{on\text{-E76A Bs}} = 0.32 \times 10^7$ $M^{-1}s^{-1}$, $k_{on\text{-Bs}} = 1.48 \times 10^7$ $M^{-1}s^{-1}$, $K_{D\text{-E76A Bs}} = 1.1$ μM, $K_{D\text{-Bs}} = 64$ nM). **(b)** Simulations of the dependence of the probability of Bs-captured events (*left panel*) and the dependence of the fractional occupancy of Bs-captured events (*right panel*) on the [Bs] value at various constant [D39A Bs] values. The model data are based on the k_{on} and K_D generated from the individual Bn-Bs and Bn-D39A Bs binding assays ($k_{on\text{-D39A Bs}} = 0.20 \times 10^7$ $M^{-1}s^{-1}$, $k_{on\text{-Bs}} = 1.48 \times 10^7$ $M^{-1}s^{-1}$, $K_{D\text{-D39A Bs}} = 168$ μM, $K_{D\text{-Bs}} = 64$ nM). **(c)** Simulations for the dependence of the probability of D39A Bs-captured events (*left panel*) and the dependence of the fractional occupancy of D39A Bs-captured events (*right panel*) on the [D39A Bs] value at various constant [Bs] values. The model data are based on the k_{on} and K_D generated from the individual Bn-Bs and Bn-D39A Bs binding assays ($k_{on\text{-D39A Bs}} = 0.20 \times 10^7$ $M^{-1}s^{-1}$, $k_{on\text{-Bs}} = 1.48 \times 10^7$ $M^{-1}s^{-1}$, $K_{D\text{-D39A Bs}} = 168$ μM, $K_{D\text{-Bs}} = 64$ nM).

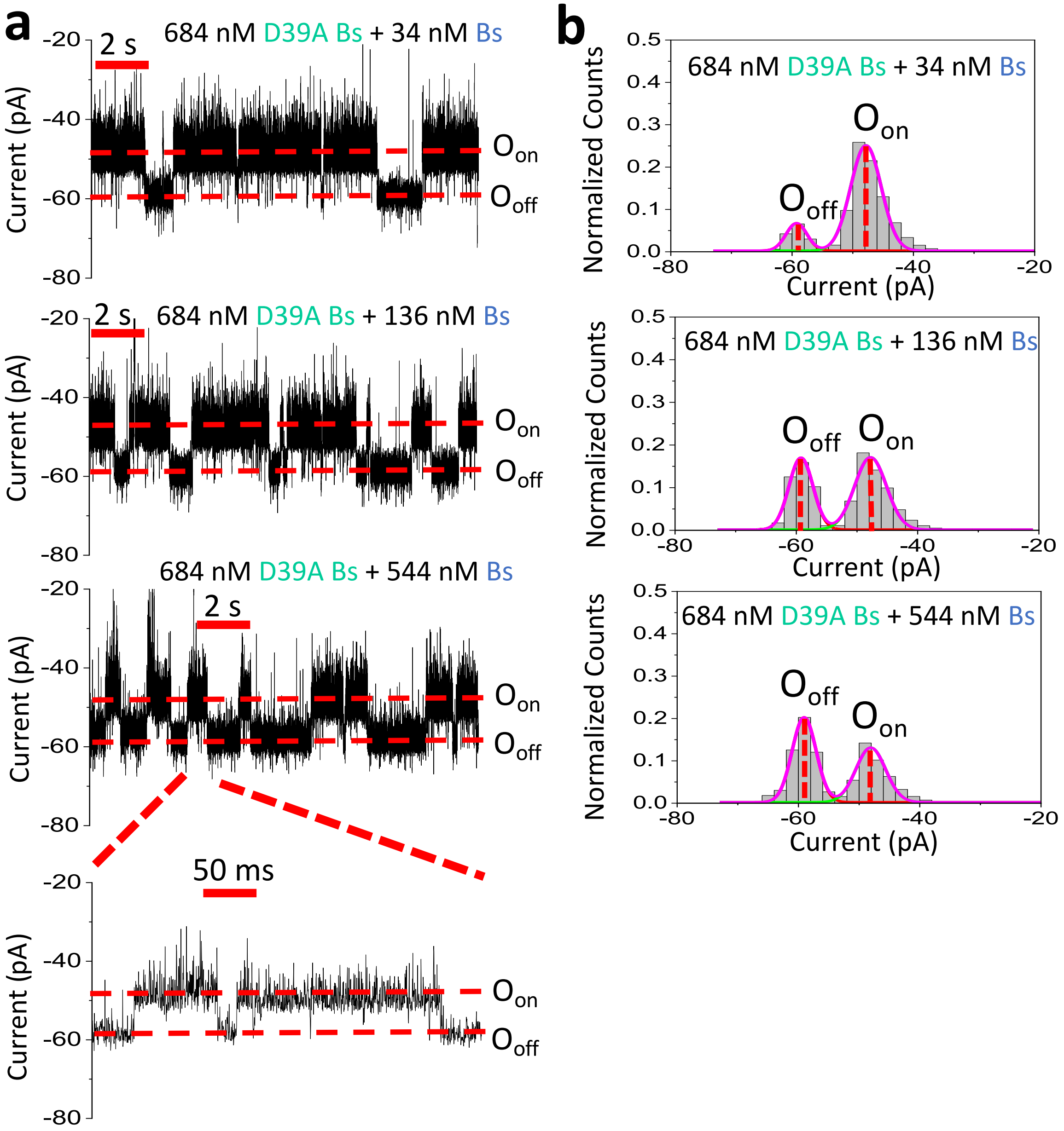

<u>Supplementary Figure S10.</u> Competitive PPIs with weakly and strongly binding interactions. (a) Representative single-channel electrical traces, which were filtered at 1 kHz using a low-pass 8-pole Bessel filter, are provided for various binary mixtures of weakly and strongly binding protein ligands. These binary mixtures contained 684 nM D39A Bs and a varying concentration of Bs added to the *cis* side of the chamber. The O_{on} and O_{off} levels correspond to the ligand-released and ligand-captured substates, respectively. The applied transmembrane potential was -40 mV. These single-channel electrical traces are representative over a subset of *n* = 3 distinct nanopores. The other recording conditions are indicated in **Experimental section**. **(b)** Corresponding normalized all-point current amplitude histograms are provided for various binary mixtures of D39A Bs and Bs. Maxima of the peaks of the current amplitude represent the substates "off" (ligand-captured event, O_{off}, *left*) and "on" (ligand-released event, O_{on}, *right*).

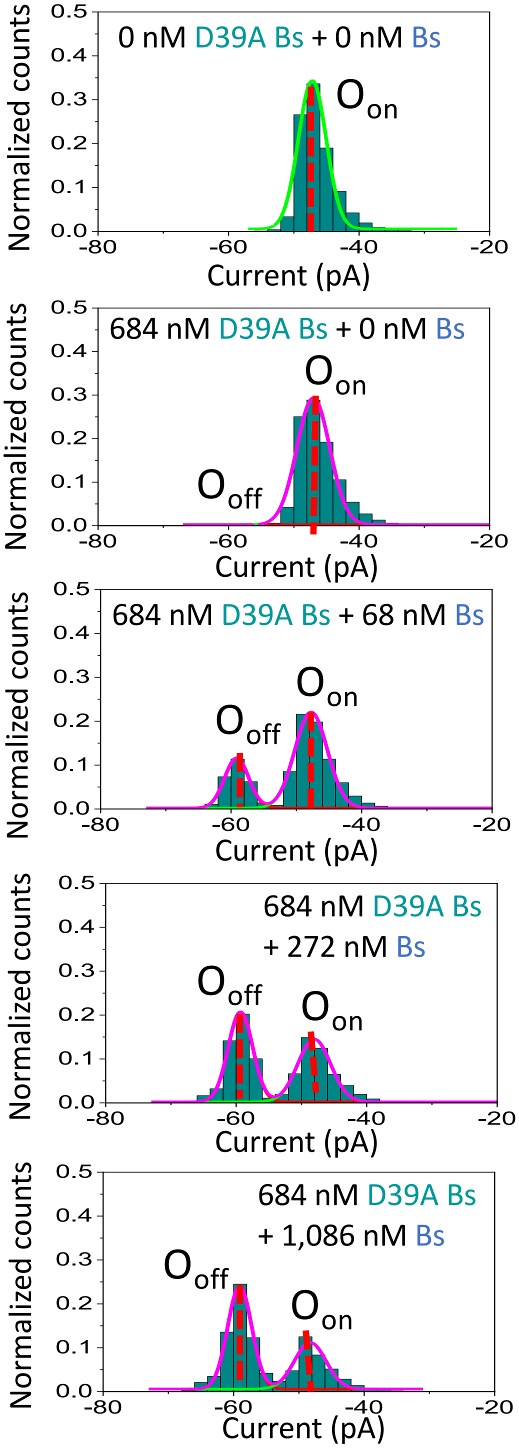

Supplementary Figure S11. Normalized all-point current amplitude histograms are provided for various binary mixtures of D39A Bs and Bs. Maxima of the peaks of the current amplitude represent the substates "off" (ligand-captured event, O_{off}, *left*) and "on" (ligand-released event, O_{on}, *right*). On the top panel, the green line corresponds to the fit of the O_{on} substate. On the other panels, the green and red lines correspond to the fits of the O_{on} and O_{off} substates, respectively. The magenta line is the cumulative fit.

<u>Supplementary Table S9.</u> The mean values of the current amplitudes represent the substates "off" (ligand-captured binding event, O_{off}) and "on" (ligand-released binding event, O_{on}) according to Figure 4b and Supplementary Figure S7b. The binary mixture contained 684 nM D39A Bs and varying [Bs] added to the *cis* side. The other recording conditions are indicated in **Experimental section**. Values are mean ± s.e.m. from a representative single-channel electrical trace.

**[D39A Bs] (nM)**	**[Bs] (nM)**	**O_{on} (pA)**	**O_{off} (pA)**
0	0	-47.2 ± 0.1	NA
684	0	-47.0 ± 0.1	-57.8 ± 6.1
684	34	-47.8 ± 0.1	-59.3 ± 0.4
684	68	-47.7 ± 0.1	-59.1 ± 0.2
684	136	-47.8 ± 0.2	-59.2 ± 0.1
684	272	-48.1 ± 0.1	-59.3 ± 0.1
684	544	-48.0 ± 0.1	-59.1 ± 0.1
684	1,086	-48.3 ± 0.2	-59.1 ± 0.1
Mean*	NA	-47.7 ± 0.4	-59.0 ± 0.5

NA stands for not applicable.
*This line indicates data directly averaged from data collected at different [Bs] values.

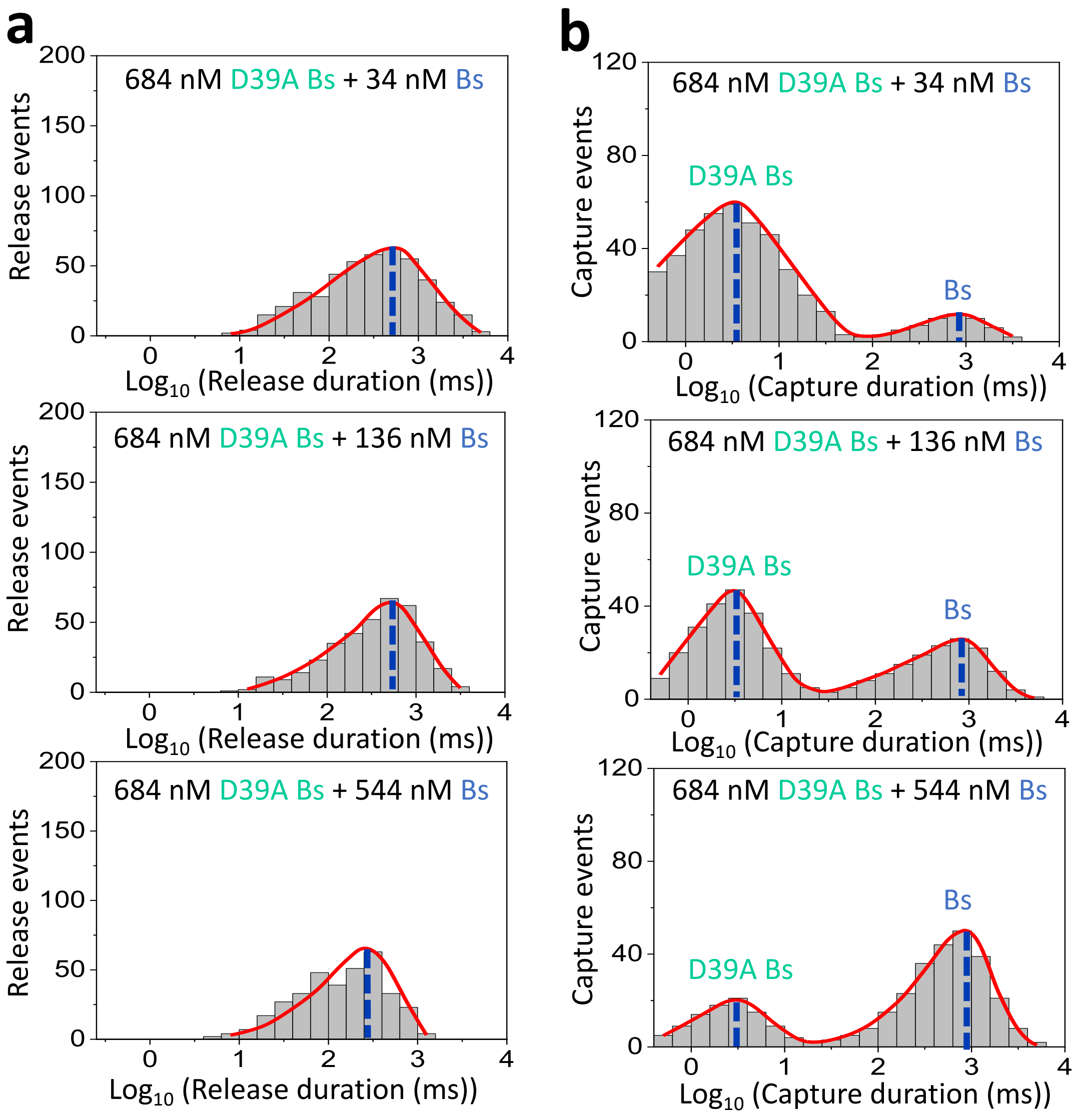

<u>Supplementary Figure S12.</u> Semilogarithmic event duration histograms resulting from competitive PPIs with weakly and strongly binding interactions. (a) Representative semilogarithmic event duration histograms of ligand-released events at various [Bs] values. 684 nM weak-affinity D39A Bs was added to the *cis* side. The τ_{on} release durations (mean ± s.e.m.) from these histograms were 603 ± 33 ms (the number of events: $N = 453$), 503 ± 26 ms ($N = 374$), and 261 ± 17 ms ($N = 350$) at 34 nM Bs, 136 nM Bs, and 544 nM Bs, respectively. (**b**) Representative semilogarithmic event duration histograms of ligand-captured events at various [Bs] values. 684 nM weak-affinity D39A Bs was added to the *cis* side. The τ_{off} capture durations (mean ± s.e.m.) from these histograms were 3.2 ± 0.2 ms and 832 ± 35 ms (the number of events: $N = 453$), 3.0 ± 0.1 ms and 841 ± 38 ms ($N = 374$), and 2.8 ± 0.1 ms and 838 ± 29 ms ($N = 350$), at 34 nM Bs, 136 nM Bs, and 544 nM Bs, respectively. The other recording conditions are indicated in **Experimental section**.

<u>Supplementary Table S10.</u> Capture (τ_{off}) and release (τ_{on}) durations of the binding events in the presence of a binary mixture of weak- and strong-affinity protein ligands. 684 nM weak-affinity D39A Bs was added to the *cis* side of the chamber. The binary mixture also encompassed a varying strong-affinity [Bs]. The k_{off} are the individual dissociation rate constants of the weak- and strong-affinity interactions. The maximum likelihood method (MLM)[2] and logarithm likelihood ratio (LLR)[3-5] tests were used to fit event duration histograms. These methods were used to determine the number of statistically significant subpopulations best represented by the data. The other recording conditions are indicated in **Experimental section**. Values are mean ± s.d. using $n = 3$ nanopores.

[Bs] (nM)	$\tau_{off\text{-}Bs}$ (ms)	$\tau_{off\text{-}D39A\ Bs}$ (ms)	τ_{on} (ms)	$k_{off\text{-}Bs}$ (s^{-1})	$k_{off\text{-}D39A\ Bs}$ (s^{-1})
0	NA	3.1 ± 0.2	752 ± 62	NA	327 ± 22
34	852 ± 52	2.9 ± 0.3	635 ± 55	1.18 ± 0.07	343 ± 32
68	841 ± 36	3.0 ± 0.3	545 ± 15	1.19 ± 0.05	340 ± 36
136	850 ± 44	2.8 ± 0.2	509 ± 14	1.18 ± 0.06	363 ± 26
272	854 ± 26	2.9 ± 0.4	348 ± 27	1.17 ± 0.04	349 ± 41
544	843 ± 44	2.8 ± 0.4	273 ± 34	1.19 ± 0.06	355 ± 46
1,086	840 ± 39	2.8 ± 0.3	192 ± 34	1.19 ± 0.06	359 ± 41
Mean*	846 ± 6	2.9 ± 0.1	NA	1.18 ± 0.01	351 ± 9

NA stands for not applicable.

*This line indicates data directly averaged from data collected at different [Bs] values.

<u>Supplementary Table S11.</u> Release durations of individual binding events in the presence of a binary mixture of weak- and strong-affinity protein ligands. 684 nM weak-affinity D39A Bs was added to the *cis* side of the chamber. The binary mixture of ligands also encompassed a varying strong-affinity [Bs]. $\tau_{on\text{-}D39\ Bs} = \tau_{on} / P_{D39\ Bs}$, where $P_{D39\ Bs}$ is the event probability of the D39 Bs-captured events (**Eqn. (1)**). $\tau_{on\text{-}Bs} = \tau_{on} / P_{Bs}$, where P_{Bs} is the event probability of the Bs-captured events (**Eqn. (1)**). Here, τ_{on} are mean values of the single-exponential distributions of ligand-released duration histograms (**Supplementary Table S10**). The k_{on} and K_D are the individual rate constants of association of the weak- and strong-affinity interactions and the equilibrium dissociation constant, respectively. The other recording conditions are indicated in **Experimental section**. The maximum likelihood method (MLM)[2] and logarithm likelihood ratio (LLR)[3-5] tests were used to fit event duration histograms. These methods were used to determine the number of statistically significant subpopulations best represented by the data. Values are mean ± s.d. using $n = 3$ independently reconstituted nanopores.

[Bs] (nM)	$\tau_{on\text{-}Bs}$ (ms)	$\tau_{on\text{-}D39A\ Bs}$ (ms)	$k_{on\text{-}Bs}$ ($10^7\ M^{-1}s^{-1}$)	$k_{on\text{-}D39A\ Bs}$ ($10^7\ M^{-1}s^{-1}$)	$K_{D\text{-}Bs}$ (nM)	$K_{D\text{-}D39A\ Bs}$ (μM)
0	NA	752 ± 62	NA	0.20 ± 0.02	NA	168 ± 24
34	4,921 ± 297	729 ± 67	0.60 ± 0.04	0.20 ± 0.02	197 ± 23	171 ± 24
68	2,463 ± 112	701 ± 27	0.60 ± 0.03	0.21 ± 0.01	199 ± 3	163 ± 11
136	1,324 ± 202	834 ± 40	0.56 ± 0.08	0.18 ± 0.01	213 ± 43	207 ± 13
272	634 ± 32	775 ± 105	0.58 ± 0.03	0.19 ± 0.03	202 ± 8	187 ± 46
544	387 ± 34	940 ± 223	0.48 ± 0.04	0.16 ± 0.04	250 ± 14	233 ± 83
1,086	241 ± 40	960 ± 252	0.39 ± 0.07	0.16 ± 0.04	311 ± 46	240 ± 90
Mean*	NA	NA	0.53 ± 0.08	0.18 ± 0.02	229 ± 45	200 ± 32

NA stands for not applicable.

*This line indicates data directly averaged from data collected at different [Bs] values.

<u>Supplementary Table S12.</u> The probability (*P*) and fractional occupancy (*F*) of Bs-captured binding events in presence of a binary mixture of weak- and strong-affinity protein ligands. *O* is the occupancy of the Bn binding site. 684 nM weak-affinity D39A Bs was added to the *cis* side of the chamber. The binary mixture also encompassed a varying strong-affinity [Bs]. The other recording conditions are indicated in **Experimental section**. Values are mean ± s.e.m. from a single representative experiment.

[Bs] (nM)	P_{Bs}	$P_{D39A\ Bs}$	F_{Bs}	$F_{D39A\ Bs}$	O^{Mod}	O^{Exp}
0	0	1	0	1	0.004	0.005
34	0.122 ± 0.009	0.878 ± 0.009	0.973 ± 0.002	0.027 ± 0.002	0.341	0.148
68	0.212 ± 0.013	0.788 ± 0.013	0.988 ± 0.001	0.012 ± 0.001	0.508	0.254
136	0.398 ± 0.014	0.602 ± 0.014	0.995 ± 0.001	0.005 ± 0.001	0.673	0.401
272	0.568 ± 0.021	0.432 ± 0.021	0.997 ± 0.001	0.003 ± 0.001	0.804	0.586
544	0.728 ± 0.018	0.272 ± 0.018	0.999 ± 0.001	0.001 ± 0.001	0.892	0.700
1,086	0.811 ± 0.012	0.189 ± 0.012	0.999 ± 0.000	0.001 ± 0.000	0.943	0.808

<u>Supplementary Table S13.</u> The probability (*P*) and fractional occupancy (*F*) of Bs-captured binding events in presence of a binary mixture of weak- and strong-affinity protein ligands. *O* is the occupancy of the Bn binding site. 684 nM weak-affinity D39A Bs was added to the *cis* side of the chamber. The binary mixture also encompassed a varying strong-affinity [Bs]. The other recording conditions are indicated in **Experimental section**. Values are mean ± s.d. using $n = 3$ independently reconstituted nanopores.

[Bs] (nM)	P_{Bs}	$P_{D39A\ Bs}$	F_{Bs}	$F_{D39A\ Bs}$	O^{Mod}	O^{Exp}
0	0	1	0	1	0.004	0.004 ± 0.001
34	0.129 ± 0.006	0.871 ± 0.006	0.977 ± 0.004	0.023 ± 0.004	0.341	0.151 ± 0.015
68	0.222 ± 0.013	0.778 ± 0.013	0.988 ± 0.001	0.012 ± 0.001	0.508	0.257 ± 0.003
136	0.389 ± 0.046	0.611 ± 0.046	0.995 ± 0.001	0.005 ± 0.001	0.673	0.394 ± 0.047
272	0.549 ± 0.033	0.451 ± 0.033	0.997 ± 0.001	0.003 ± 0.001	0.804	0.575 ± 0.010
544	0.704 ± 0.044	0.296 ± 0.044	0.9985 ± 0.0006	0.0015 ± 0.0006	0.892	0.686 ± 0.013
1,086	0.796 ± 0.031	0.204 ± 0.031	0.9991 ± 0.0003	0.0009 ± 0.0003	0.943	0.778 ± 0.026

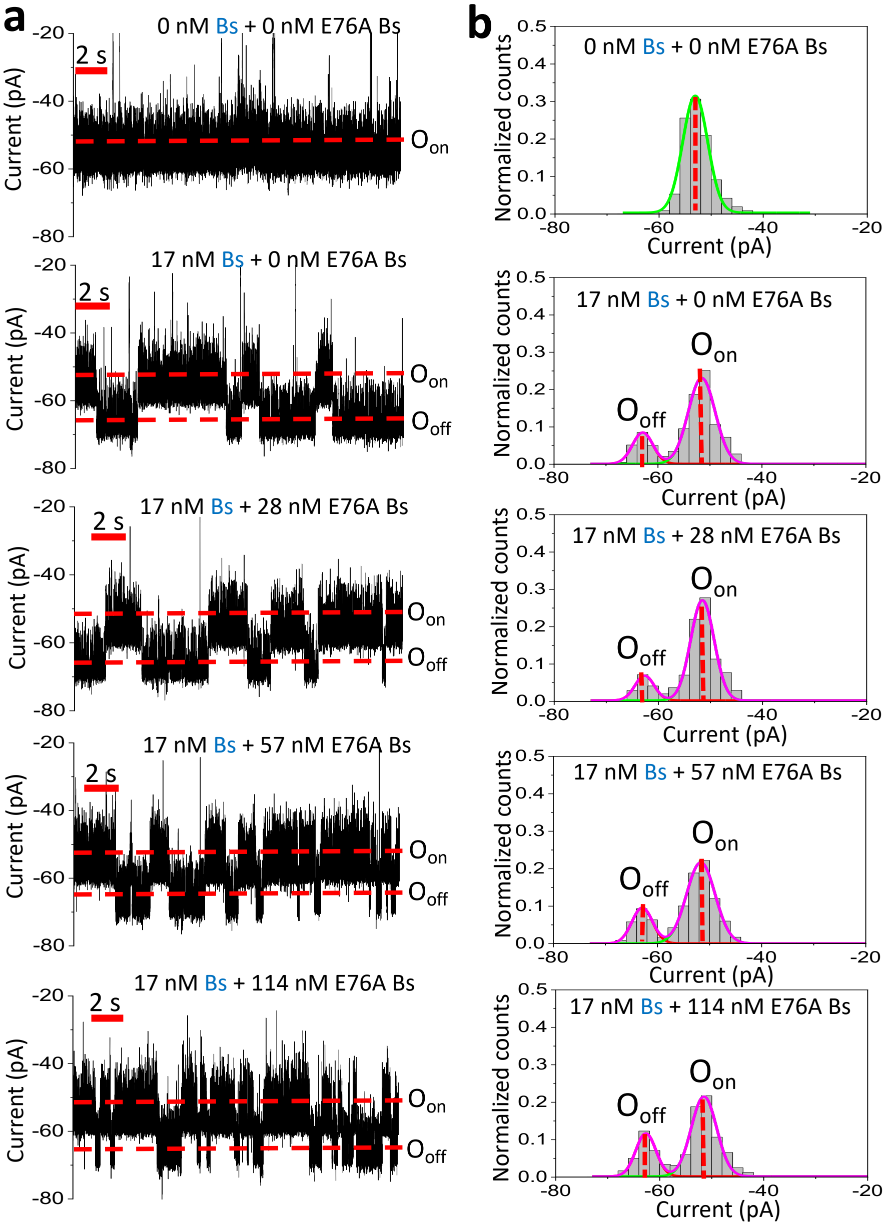

<u>Supplementary Figure S13.</u> Competitive PPIs with moderately and strongly binding interactions. (**a**) Representative single-channel electrical traces, which were filtered at 1 kHz using a low-pass 8-pole Bessel filter. The binary mixtures contained 17 nM Bs and varying [E76A Bs] added to the *cis* side. The [E76A Bs] range was 0 – 114 nM. The O_{on} and O_{off} levels correspond to the ligand-released and ligand-captured substates, respectively. The applied transmembrane potential was -40 mV. These single-channel electrical traces are representative over a subset of $n = 3$ distinct nanopores. The other recording conditions are indicated in **Experimental section**. (**b**) Corresponding normalized all-point current amplitude histograms are provided for various binary mixtures of Bs and E76A Bs. Maxima of the peaks of the current amplitude represent the substates "off" (ligand-captured event, O_{off}, *left*) and "on" (ligand-released event, O_{on}, *right*).

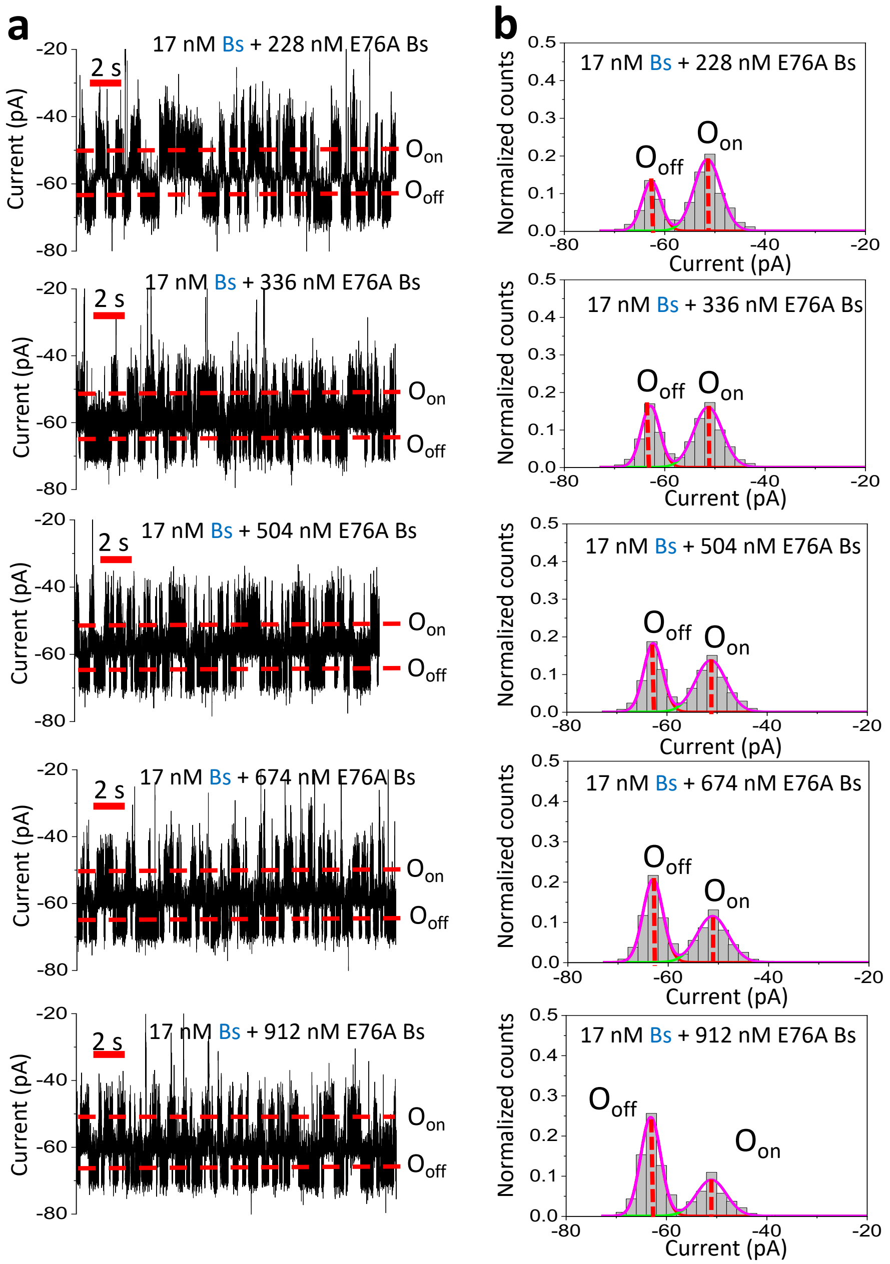

<u>Supplementary Figure S14.</u> Competitive PPIs with moderately and strongly binding interactions. (**a**) Representative single-channel electrical traces, which were filtered at 1 kHz using a low-pass 8-pole Bessel filter. The binary mixtures contained 17 nM Bs and varying [E76A Bs] added to the *cis* side. The [E76A Bs] range was 228 – 912 nM. The O_{on} and O_{off} levels correspond to the ligand-released and ligand-captured substates, respectively. The applied transmembrane potential was -40 mV. These single-channel electrical traces are representative over a subset of $n = 3$ distinct nanopores. The other recording conditions are indicated in **Experimental section**. (**b**) Corresponding normalized all-point current amplitude histograms are provided for various binary mixtures of Bs and E76A Bs. Maxima of the peaks of the current amplitude represent the substates "off" (ligand-captured event, O_{off}, *left*) and "on" (ligand-released event, O_{on}, *right*).

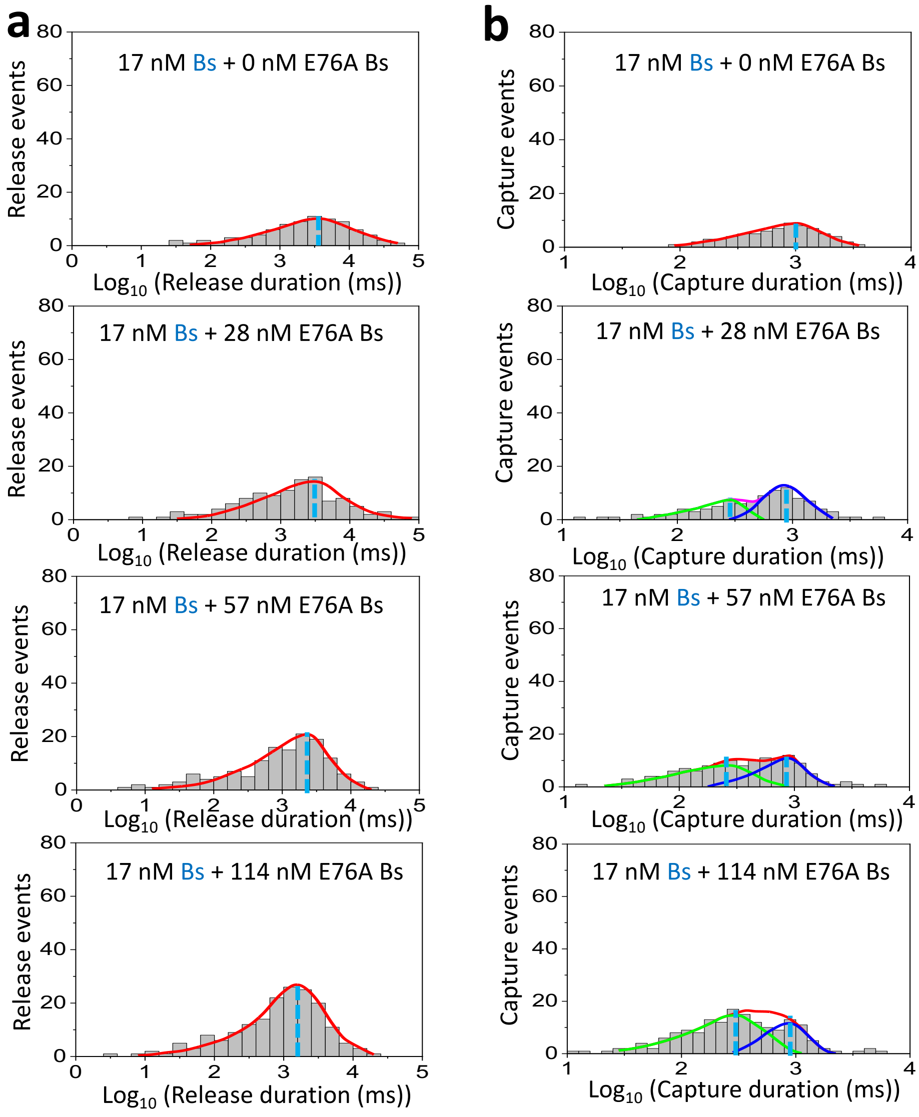

<u>Supplementary Figure S15.</u> Semilogarithmic event duration histograms resulting from competitive PPIs with strongly and moderately binding interactions. (**a**) Representative semilogarithmic event duration histograms of ligand-released events at various E76A Bs concentrations. The binary mixtures contained 17 nM strong-affinity Bs and varying moderate-affinity E76A Bs. The τ_{on} release durations (mean ± s.e.m.) from these histogram fits were 3,818 ± 133 ms (the number of events: N = 82), 3,098 ± 67 ms (N = 107), 2,410 ± 41 ms (N = 139), and 1,625 ± 36 ms (N = 175) at 0 nM E76A Bs, 28 nM E76A Bs, 57 nM E76A Bs, and 114 nM E76A Bs, respectively. (**b**) Representative semilogarithmic event duration histograms of ligand-captured events at various [E76A Bs] values. The τ_{off} capture durations (mean ± s.e.m.) from these histogram fits were 1,047 ± 35 ms (the number of events: N = 74) , 832 ± 28 ms and 289 ± 13 ms (N = 103), 871 ± 17 ms and 269 ± 11 ms (N = 132), and 891 ± 12 ms and 295 ± 8 ms (N = 167) at 0 nM E76A Bs, 28 nM E76A Bs, 57 nM E76A Bs, and 114 nM E76A Bs, respectively. The other recording conditions are indicated in the **Experimental section**.

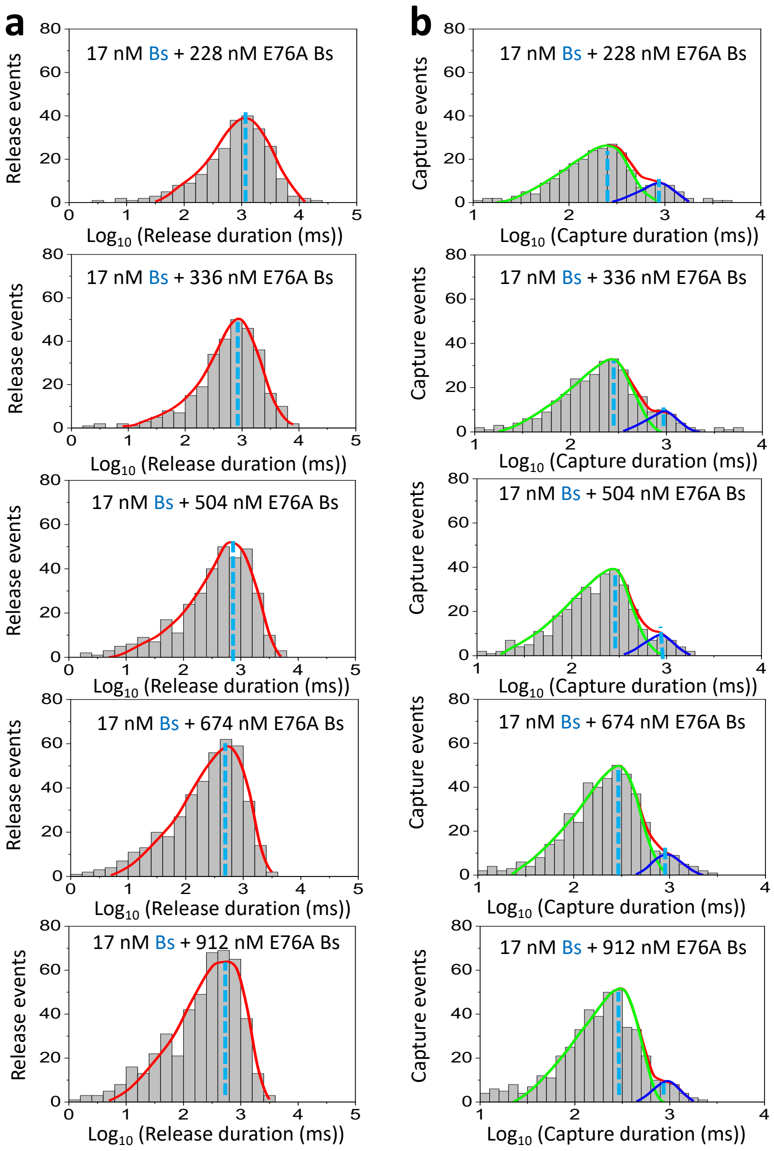

<u>Supplementary Figure S16.</u> Semilogarithmic event duration histograms resulting from competitive PPIs with strongly and moderately binding interactions. (**a**) Representative semilogarithmic event duration histograms of ligand-released events at various E76A Bs concentrations. The binary mixtures contained 17 nM strong-affinity Bs and varying moderate-affinity E76A Bs. The τ_{on} release durations (mean ± s.e.m.) from these histogram fits were 1,078 ± 28 ms (the number of events: N = 249), 861 ± 32 ms (N = 304), 679 ± 25 ms (N = 347), 509 ± 29 ms (N = 433), and 456 ± 16 ms (N = 460) at 228 nM E76A Bs, 336 nM E76A Bs, 504 nM E76A Bs, 674 nM E76A Bs, and 912 nM E76A Bs, respectively. (**b**) Representative semilogarithmic event duration histograms of ligand-captured events at various [E76A Bs] values. The τ_{off} capture durations (mean ± s.e.m.) from these histogram fits were 861 ± 25 ms and 257 ± 14 ms (the number of events: N = 248), 933 ± 34 ms and 266 ± 14 ms (N = 302), 869 ± 29 ms and 271 ± 9 ms (N = 345), 918 ± 22 ms and 289 ± 11 ms (N = 426), and 912 ± 15 ms and 302 ± 13 ms (N = 450) at 228 nM E76A Bs, 336 nM E76A Bs, 504 nM E76A Bs, 674 nM E76A Bs, and 912 nM E76A Bs, respectively. The other recording conditions are indicated in the **Experimental section**.

Supplementary Table S14. The mean values of the current amplitudes represent the substates "off" (ligand-captured binding event, O_{off}) and "on" (ligand-released binding event, O_{on}) according to Supplementary Figures S9b-S10b. The binary mixture included 17 nM Bs and varying [E76A Bs] added to the *cis* side. The other recording conditions are indicated in the **Experimental section**. Values are mean ± s.e.m. from a representative single-channel electrical trace.

[Bs] nM	[E76A Bs] nM	O_{on} (pA)	O_{off} (pA)
0	0	-53.1 ± 0.1	NA
17	0	-51.7 ± 0.1	-63.0 ± 0.2
17	28	-51.5 ± 0.1	-62.6 ± 0.3
17	57	-51.5 ± 0.1	-62.7 ± 0.2
17	114	-51.6 ± 0.1	-63.0 ± 0.3
17	228	-51.5 ± 0.1	-62.7 ± 0.1
17	336	-51.4 ± 0.1	-62.8 ± 0.1
17	504	-51.3 ± 0.1	-62.8 ± 0.1
17	674	-51.2 ± 0.1	-63.0 ± 0.1
17	912	-51.0 ± 0.1	-63.1 ± 0.1

NA stands for not applicable.

Supplementary Table S15. Ligand-captured (τ_{off}) and ligand-released (τ_{on}) durations of the binding events in the presence of a binary mixture of medium- and strong-affinity protein ligands. 17 nM strong-affinity Bs was added to the *cis* side of the chamber. The binary mixture also encompassed a varying medium-affinity [E76A Bs]. The k_{off} are the individual dissociation rate constants of the medium- and strong-affinity interactions. The other recording conditions are indicated in the **Experimental section**. The maximum likelihood method (MLM)[2] and logarithm likelihood ratio (LLR)[3-5] tests were used to fit event duration histograms. Values are mean ± s.e.m. from a single reconstituted nanopore.

[E76A Bs] (nM)	$\tau_{off\text{-}Bs}$ (ms)	$\tau_{off\text{-}E76A\ Bs}$ (ms)	τ_{on} (ms)	$k_{off\text{-}Bs}$ (s^{-1})	$k_{off\text{-}E76A\ Bs}$ (s^{-1})
0	1,047 ± 35	NA	3,818 ± 133	0.95	NA
28	832 ± 28	289 ± 13	3,098 ± 67	1.20	3.46
57	871 ± 17	269 ± 11	2,410 ± 41	1.15	3.72
114	891 ± 12	295 ± 8	1,625 ± 36	1.12	3.39
228	861 ± 25	257 ± 14	1,078 ± 28	1.16	3.89
336	933 ± 34	266 ± 14	861 ± 32	1.07	3.76
504	869 ± 29	271 ± 9	679 ± 25	1.15	3.69
674	918 ± 22	289 ± 11	509 ± 29	1.09	3.46
912	912 ± 15	302 ± 13	456 ± 16	1.10	3.31

NA stands for not applicable.

<u>Supplementary Table S16.</u> Ligand-released durations of individual binding events in the presence of a binary mixture of medium- and strong-affinity protein ligands. 17 nM Bs was added to the *cis* side of the chamber. The binary mixture of protein ligands also encompassed a varying [E76A Bs]. k_{on} and K_D are the individual rate constants of association and the equilibrium dissociation constant, respectively. The other recording conditions are indicated in the **Experimental section**. The maximum likelihood method (MLM)[2] and logarithm likelihood ratio (LLR)[3-5] tests were used to fit event duration histograms. These methods were used to determine the number of statistically significant subpopulations best represented by the data. Values are the mean from a single reconstituted nanopore.

[E76A Bs] (nM)	τ_{on-Bs} (ms)	$\tau_{on-E76A\ Bs}$ (ms)	k_{on-Bs} (10^7 $M^{-1}s^{-1}$)	$k_{on-D76A\ Bs}$ (10^7 $M^{-1}s^{-1}$)	K_{D-Bs} (nM)	$K_{D-E76A\ Bs}$ (μM)
0	3,817	NA*	1.54	NA*	62.0	NA*
28	5,181	7,705	1.14	0.46	106	0.75
57	5,347	4,389	1.10	0.40	104	0.93
114	5,244	2,355	1.12	0.37	100	0.91
228	6,966	1,275	0.84	0.34	138	1.13
336	7,130	980	0.82	0.30	130	1.24
504	6,923	753	0.85	0.26	135	1.40
674	6,773	550	0.87	0.27	125	1.28
912	6,619	490	0.89	0.22	123	1.48

*NA stands for not applicable.

<u>Supplementary Table S17.</u> The probability (*P*) and fractional occupancy (*F*) of E76A Bs-captured binding events in the presence of a binary mixture of medium- and strong-affinity protein ligands. *O* is the occupancy of the Bn binding site. 17 nM strong-affinity Bs was added to the *cis* side of the chamber. The binary mixture also encompassed a varying medium-affinity [E76A Bs]. The other recording conditions are indicated in the **Experimental section**. Values are mean ± s.e.m. from a single representative experiment.

[E76A Bs] (nM)	$P_{E76A\ Bs}$	P_{Bs}	$F_{E76A\ Bs}$	F_{Bs}	O^{Mod}	O^{Exp}
0	0	1	0	1	0.204	0.215
28	0.402 ± 0.018	0.598 ± 0.018	0.189 ± 0.011	0.811 ± 0.011	0.220	0.165
57	0.549 ± 0.020	0.451 ± 0.020	0.273 ± 0.012	0.727 ± 0.012	0.235	0.183
114	0.690 ± 0.015	0.310 ± 0.015	0.424 ± 0.015	0.576 ± 0.015	0.263	0.228
228	0.845 ± 0.013	0.155 ± 0.013	0.620 ± 0.010	0.380 ± 0.010	0.314	0.245
336	0.879 ± 0.009	0.121 ± 0.009	0.675 ± 0.009	0.325 ± 0.009	0.356	0.287
504	0.902 ± 0.011	0.098 ± 0.011	0.742 ± 0.005	0.258 ± 0.005	0.412	0.327
674	0.925 ± 0.006	0.075 ± 0.006	0.795 ± 0.010	0.205 ± 0.010	0.460	0.398
912	0.931 ± 0.005	0.069 ± 0.005	0.817 ± 0.008	0.183 ± 0.008	0.515	0.430

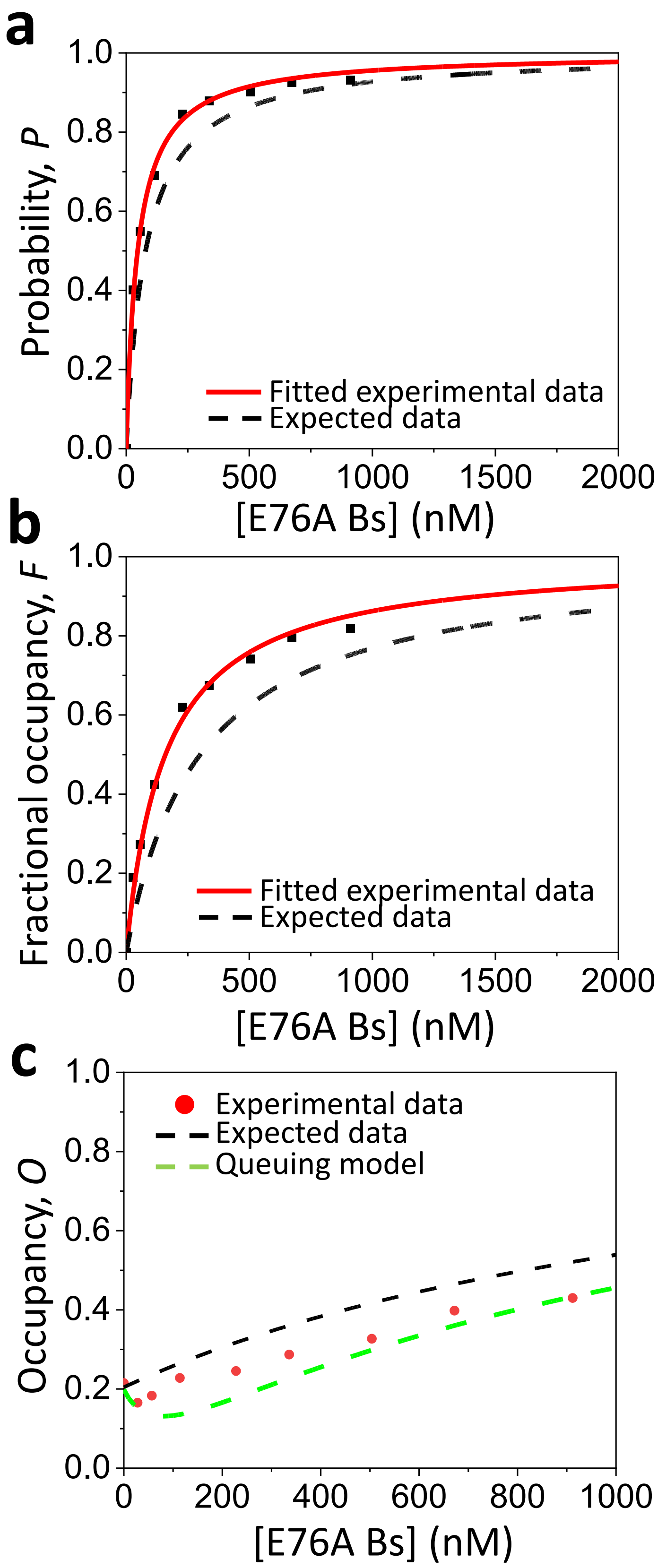

<u>Supplementary Figure S17.</u> Diagram illustrating the dependence of the probability, *P*, and fractional occupancy, *F*, and occupancy on varying [E76A Bs]. 17 nM Bs was added to the *cis* side of the chamber. [E76A Bs] was titrated to the same side in a single series of single-channel electrical recordings. **(a)** Diagram illustrating the dependence of the probability, *P*, of E76A Bs-captured binding events on the [E76A Bs] value. The black dashed line represents the model data based on the k_{on} generated from the individual Bn-Bs and Bn-E76A Bs binding assays ($k_{on\text{-E76A Bs}} = 0.32 \times 10^7$ $M^{-1}s^{-1}$, $k_{on\text{-Bs}} = 1.48 \times 10^7$ $M^{-1}s^{-1}$). The red continuous line represents the fit of experimental data. Using the fit of experimental data **(eqn. (1))**, the k_{on} for Bs and E76A Bs were $(1.28 \pm 0.04) \times 10^7$ $M^{-1}s^{-1}$ and $(0.42 \pm 0.01) \times 10^7$ $M^{-1}s^{-1}$, respectively. **(b)** Diagram illustrating the dependence of the fractional occupancy, *F*, of E76A Bs-captured binding events on the [E76A Bs] value. The black dashed line represents the model data based on the K_D generated from the individual Bn-Bs and Bn-E76A Bs binding assays ($K_{D\text{-Bs}}$ = 64 nM and $K_{D\text{-E76A Bs}}$ = 1.1 μM). The red continuous line represents the fit of experimental data. Using the fit of experimental data **(eqn. (2))**, the K_D for Bs and E76A Bs were 97 ± 6 nM and 0.98 ± 0.02 nM, respectively. **(c)** Diagram illustrating the dependence of the occupancy, *O*, of the Bn binding site at various [E76A Bs] values. The experimental data of $P_{\text{E76A Bs}}$ and $F_{\text{E76A Bs}}$ are listed in **Supplementary Table S17**. The expected and experimental values of the receptor occupancy are also listed in **Supplementary Table S17**.

REFERENCES

1. Mayse, L. A.; Imran, A.; Larimi, M. G.; Cosgrove, M. S.; Wolfe, A. J.; Movileanu, L., Disentangling the recognition complexity of a protein hub using a nanopore. *Nature Commun.* **2022,** *13* (1), 978.

2. Colquhoun, D.; Sigworth, F. J., Fitting and statistical analysis of single-channel records. In *Single-channel recording*, 2nd ed.; Sackmann, B., Neher,E., Ed. Plenum Press: New York, 1995; pp 483-587.

3. McManus, O. B.; Blatz, A. L.; Magleby, K. L., Sampling, Log Binning, Fitting, and Plotting Durations of Open and Shut Intervals From Single Channels and the Effects of Noise. *Pflugers Arch.* **1987,** *410* (4-5), 530-553.

4. McManus, O. B.; Magleby, K. L., Kinetic States and Modes of Single Large-Conductance Calcium-Activated Potassium Channels in Cultured Rat Skeletal-Muscle. *J. Physiol. (Lond.)* **1988,** *402*, 79-120.

5. Couoh-Cardel, S.; Hsueh, Y. C.; Wilkens, S.; Movileanu, L., Yeast V-ATPase Proteolipid Ring Acts as a Large-conductance Transmembrane Protein Pore. *Sci. Rep.* **2016,** *6*, 24774.
